# TACE-HAIC plus targeted therapy and immune checkpoint inhibitors for unresectable hepatocellular carcinoma: a systematic review and meta-analysis of TACE- and HAIC-based comparisons

**DOI:** 10.3389/fonc.2026.1942009

**Published:** 2026-10-23

**Authors:** Peizhuo Li, Hao Li, Boyu Mei, Simin Feng, Huanzhang Niu

**Affiliations:** Department of Interventional Radiology, The First Affiliated Hospital of Henan University of Science and Technology, Luoyang, Henan, China

**Keywords:** HAIC, hepatocellular carcinoma, immune checkpoint inhibitors, TACE, targeted therapy

## Abstract

**Background:**

Against the backdrop of targeted therapy and immune checkpoint inhibitors (ICIs), the additional benefit of combining TACE and HAIC remains uncertain in unresectable hepatocellular carcinoma (uHCC). We separately evaluated TACE-based and HAIC-based comparator strategies.

**Methods:**

CNKI, the Cochrane Library, Embase, PubMed, SinoMed, VIP, Wanfang, and Web of Science were searched from inception to 19 August 2026. Overall survival (OS) was designated as the sole primary efficacy outcome; progression-free survival (PFS), objective response rate (ORR), disease control rate (DCR), and complete response (CR) were secondary efficacy outcomes. Comparator-specific random-effects meta-analyses were performed. Risk of bias for survival outcomes was assessed using ROBINS-I, and certainty of evidence across outcomes was evaluated using GRADE.

**Results:**

In all, 14 retrospective cohort studies were included. The TACE-based and HAIC-based OS analyses included nine and five comparisons involving 1,510 and 572 participants, respectively. Compared with TACE plus targeted therapy and ICIs, TACE-HAIC plus targeted therapy and ICIs was associated with a lower hazard of death (HR = 0.59, 95% CI: 0.47–0.75) and progression or death (HR = 0.65, 95% CI: 0.55–0.78), and with higher ORR (RR = 1.34, 95% CI: 1.19–1.52), DCR (RR = 1.14, 95% CI: 1.07–1.21), and CR (RR = 2.17, 95% CI: 1.46–3.21). Against an HAIC-based comparator, TACE-HAIC was associated with a lower hazard of death (HR = 0.56, 95% CI: 0.43–0.72) and progression or death (HR = 0.64, 95% CI: 0.48–0.86), and with a higher ORR (RR = 1.24, 95% CI: 1.03–1.50), whereas DCR and CR showed no clear between-group differences. Overall grade ≥3 TRAEs were imprecisely estimated in both comparisons (TACE-based: RR = 1.16, 95% CI: 0.81–1.67; HAIC-based: RR = 1.27, 95% CI: 0.92–1.74).

**Conclusions:**

TACE-HAIC plus targeted therapy and ICIs was associated with improved survival and tumor-response outcomes compared with a TACE-based regimen. Against an HAIC-based regimen, associations favored TACE-HAIC for OS, PFS, and ORR in the main analyses, although the PFS and ORR findings were less robust in sensitivity analyses. Available evidence is insufficient to determine whether TACE-HAIC increases the overall risk of grade ≥3 TRAEs. The certainty of evidence was low to very low, and prospective validation is required.

**Systematic review registration:**

https://www.crd.york.ac.uk/PROSPERO/view/CRD420261286742, identifier CRD420261286742.

## Introduction

Hepatocellular carcinoma (HCC) ranks sixth in cancer incidence and third in cancer-related mortality globally (1). More than half of patients are diagnosed at an intermediate or advanced stage because of the insidious onset and lack of obvious early symptoms. Consequently, many patients are no longer candidates for curative treatments such as surgical resection or ablation and present with unresectable hepatocellular carcinoma (uHCC) (2). To improve outcomes in uHCC, combination strategies integrating locoregional therapies with systemic targeted therapy and immune checkpoint inhibitors (ICIs) are being actively explored (3).

Among locoregional therapies, TACE, which blocks the tumor blood supply through chemoembolization and induces ischemic necrosis, is globally recognized as a major transarterial treatment modality for uHCC (4). HAIC, which involves the continuous infusion of chemotherapeutic agents through the hepatic artery to achieve high local drug concentrations, has gained particular attention in the Asia-Pacific region for uHCC with portal vein tumor thrombosis (PVTT) or high tumor burden (HTB) (5). TACE is limited by hypoxia-induced resistance and incomplete embolization, whereas HAIC lacks the immediate tumor-debulking effect provided by physical embolization. The sequential or combined use of the two modalities may therefore provide complementary effects (6, 7).

Targeted therapy and ICIs have substantially reshaped the treatment landscape for uHCC (8). The pivotal phase III IMbrave150 trial showed that atezolizumab plus bevacizumab improved PFS and OS relative to sorafenib (9), and the CARES-310 trial further demonstrated the first-line efficacy of camrelizumab plus rivoceranib (10). Current ASCO and ESMO guidelines include ICI-based systemic therapy among key first-line options for uHCC (11, 12). Although these regimens improve survival, intrahepatic progression remains an important clinical problem, supporting continued investigation of locoregional-systemic combinations.

The EMERALD-1 trial demonstrated improved PFS with TACE plus durvalumab and bevacizumab relative to TACE alone (13), and LEAP-012 showed a PFS advantage with lenvatinib and pembrolizumab plus TACE (14). HAIC-based combinations with targeted therapy and ICIs have also shown encouraging activity in advanced uHCC (15, 16). TACE- or HAIC-based systemic combinations can improve tumor control but may increase treatment-related toxicity (17, 18). In patients with HTB, large or multifocal intrahepatic tumors, or PVTT, a single transarterial modality may be insufficient to simultaneously provide rapid tumor debulking, sustained regional chemotherapy, and control of residual lesions (19). TACE-HAIC combined with targeted therapy and ICIs has therefore emerged as an intensified treatment strategy.

To date, 14 retrospective studies have compared TACE-HAIC plus targeted therapy and ICIs with either TACE-based or HAIC-based targeted therapy plus ICIs, with inconsistent findings (20–33). Therefore, we separately evaluated the efficacy and safety of TACE-HAIC plus targeted therapy and ICIs versus TACE plus targeted therapy and ICIs and versus HAIC plus targeted therapy and ICIs in patients with unresectable HCC.

## Methods

### Study registration and reporting

This study was registered in PROSPERO (Registration No. CRD420261286742) and was conducted and reported in accordance with the PRISMA 2020 Statement (34). A separate full protocol was not prepared. Amendments to the registered methods are documented in the PROSPERO version history.

### Literature search

CNKI, the Cochrane Library, Embase, PubMed, SinoMed, VIP, Wanfang, and Web of Science were searched from inception to 19 August 2026. Controlled vocabulary and free-text terms for hepatocellular carcinoma, transarterial chemoembolization (TACE), and hepatic arterial infusion chemotherapy (HAIC) were combined using Boolean operators, including direct TACE-HAIC terminology where supported by the database. Targeted therapy and immune checkpoint inhibitors were assessed as eligibility criteria during study screening rather than included as mandatory search concepts. No study-design filter was applied (**Supplementary Table S1**).

### Eligibility criteria

#### Inclusion criteria

1. Study population: patients with HCC at BCLC stage B/C or with other clearly defined unresectable characteristics; 

2. Intervention: TACE-HAIC plus targeted therapy and ICIs; 

3. Comparator: TACE plus targeted therapy and ICIs or HAIC plus targeted therapy and ICIs; 

4. Outcomes: OS, PFS, ORR, DCR, CR, overall grade ≥3 treatment-related adverse events (TRAEs), or specific adverse events (AEs); and 

5. Study design: randomized controlled trials or comparative cohort studies. Targeted therapy included TKIs, anti-VEGF/VEGFR agents, and other anti-angiogenic agents, whereas immunotherapy was restricted to ICIs, primarily PD-1 or PD-L1 inhibitors.

#### Exclusion criteria

1. Animal or basic experimental studies, case reports, case series, commentaries, conference abstracts only, reviews, meta-analyses, or unpublished studies; 

2. studies with ineligible comparators or without extractable outcome data; 

3. duplicate reports or overlapping patient populations within the same comparator-specific synthesis; 

4. reports without full text; and 

5. single-arm studies without an eligible control group.

### Data extraction and risk-of-bias assessment

Two investigators independently screened the literature and extracted study characteristics, treatment regimens, follow-up, and outcome data, with disagreements resolved by discussion or consultation with a third investigator. For OS and PFS, directly reported HRs and 95% CIs were preferentially extracted; when unavailable, survival estimates were reconstructed from Kaplan-Meier curves using the Guyot method (35). Binary outcomes were extracted from reported arm-level event counts. Risk of bias for the survival outcomes was assessed using ROBINS-I (36), with the Newcastle-Ottawa Scale retained as a supplementary descriptive assessment (37), and certainty of evidence was evaluated using GRADE (38). Risk-of-bias and certainty assessments were performed independently by two investigators, with disagreements resolved by discussion or consultation with a third investigator.

### Outcomes and statistical analysis

Overall survival (OS) was designated as the sole primary efficacy outcome, whereas PFS, ORR, DCR, and CR were secondary efficacy outcomes. Overall grade ≥3 TRAEs were the principal safety outcome, and individual AEs were exploratory safety outcomes. Statistical analyses were performed using R version 4.5.2. TACE-based and HAIC-based comparators were analyzed separately. HRs and 95% CIs were pooled for OS and PFS, whereas RRs and 95% CIs were pooled for binary outcomes. Random-effects models were used throughout, with between-study variance estimated using restricted maximum likelihood (REML). Heterogeneity was assessed using Cochran’s Q, I², and τ². Individual AEs were pooled when informative comparative data were available from at least three studies. Sensitivity analyses included leave-one-out analyses for efficacy outcomes; for survival outcomes, additional analyses were performed after excluding studies at serious overall risk of bias according to ROBINS-I. Meta-regression and assessment of publication bias using funnel plots or regression-based tests were not performed because fewer than 10 studies were available for each comparator-specific outcome. Two-sided P < 0.05 was considered statistically significant.

## Results

### Study selection

The database searches identified 3,612 records. After removal of 1,417 duplicate records, 2,195 unique records underwent title and abstract screening. Of these, 2,094 were excluded, leaving 101 reports for detailed eligibility assessment. A total of 87 reports were excluded: 24 for ineligible interventions, 15 for ineligible comparators, 15 for the absence of an eligible comparative arm, 22 for an ineligible publication type, 3 for ineligible populations, 3 for overlapping or superseded cohorts, and 5 because an eligible comparator-specific subgroup or outcome was not separately extractable. In all, 14 studies were included (**Figure 1**; **Supplementary Table S9**).

**Figure 1:**
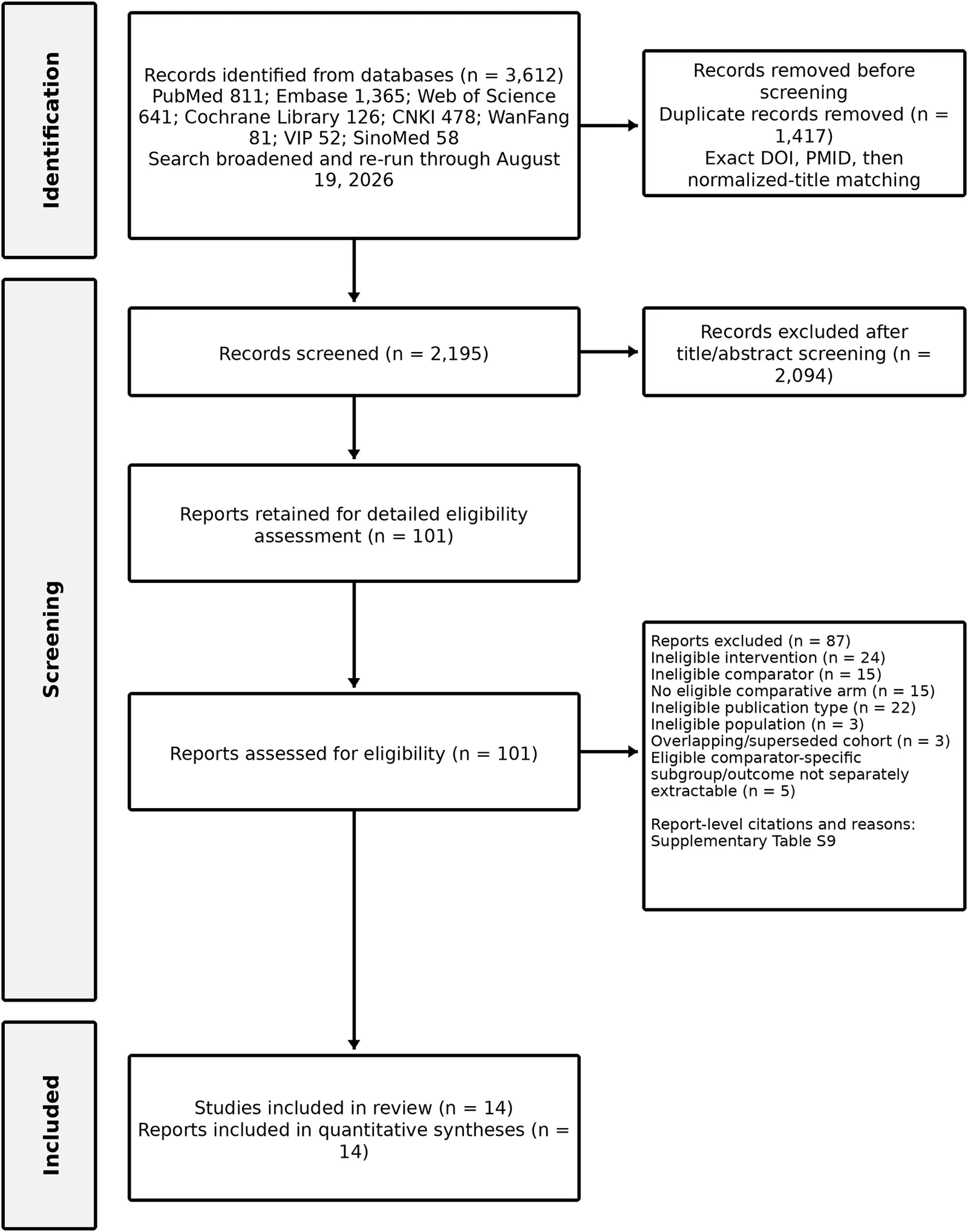
PRISMA 2020 flow diagram for study selection.

### Baseline study characteristics

The 14 eligible studies were published between 2024 and 2026 and were all conducted in China. Six studies used propensity-score matching (PSM) for the included comparisons, two used inverse probability of treatment weighting (IPTW), one unmatched cohort contributed a multivariable Cox-adjusted OS estimate, and five studies contributed unadjusted direct treatment comparisons for at least one included outcome. The TACE-based and HAIC-based OS analyses included 1,510 and 572 participants, respectively. The participants were predominantly middle-aged or older men with Child-Pugh class A or B liver function and unresectable HCC, primarily at BCLC stage B/C or corresponding CNLC stages. The baseline characteristics and treatment regimens of the included studies are summarized in **Tables 1a** and **1b**.

**Table 1a:** Baseline characteristics of the included study populations.

Study	Group	n	Age	Male, n (%)	Child-Pugh class, n	ECOG PS, n	Tumor stage, n	PVTT/vascular invasion, n	Extrahepatic metastasis, n
Chi 2025 (20)	TACE-HAIC + targeted therapy + ICIs	24	51.5 (31-84)	22 (91.7)	A: 15; B: 9	0: 21; 1: 3	BCLC B: 0; C: 24	PVTT Yes: 24; No: 0	Yes: 3; No: 21
Chi 2025 (20)	TACE + targeted therapy + ICIs	38	57 (26-80)	34 (89.5)	A: 29; B: 9	0: 33; 1: 5	BCLC B: 0; C: 38	PVTT Yes: 38; No: 0	Yes: 13; No: 25
Chi 2025 (20)	HAIC + targeted therapy + ICIs	12	53.5 (45-80)	11 (91.7)	A: 8; B: 4	0: 11; 1: 1	BCLC B: 0; C: 12	PVTT Yes: 12; No: 0	Yes: 5; No: 7
Huang 2024 (21)	TACE-HAIC + targeted therapy + ICIs	63	≤60: 43; >60: 20	57 (90.5)	A: 39; B: 24	0: 28; 1: 35	BCLC B: 26; C: 37	Venous cancer thrombosis Yes: 24; No: 39	Yes: 24; No: 39
Huang 2024 (21)	TACE + targeted therapy + ICIs	60	≤60: 34; >60: 26	52 (86.7)	A: 41; B: 19	0: 31; 1: 29	BCLC B: 31; C: 29	Venous cancer thrombosis Yes: 22; No: 38	Yes: 21; No: 39
Feng 2026 (22)	TACE-HAIC + targeted therapy + ICIs	107	≤60: 58; >60: 49	95 (88.8)	A: 81; B: 26	0: 60; 1: 47	BCLC B: 31; C: 76	PVTT Yes: 36; No: 71	Yes: 34; No: 73
Feng 2026 (22)	TACE + targeted therapy + ICIs	107	≤60: 55; >60: 52	95 (88.8)	A: 83; B: 24	0: 66; 1: 41	BCLC B: 36; C: 71	PVTT Yes: 36; No: 71	Yes: 33; No: 74
Li 2025 (23)^b^	TACE-HAIC + targeted therapy + ICIs	154	<60: 112; ≥60: 42	140 (90.9)	NR	0: 109; 1: 45	BCLC B: 29; C: 125	Vascular invasion Yes: 103; No: 51	Yes: 73; No: 81
Li 2025 (23)^b^	TACE + targeted therapy + ICIs	305	<60: 204; ≥60: 101	269 (88.2)	NR	0: 233; 1: 72	BCLC B: 136; C: 169	Vascular invasion Yes: 128; No: 177	Yes: 95; No: 210
Liu 2025 (24)	TACE-HAIC + targeted therapy + ICIs	50	57 (53-63)	43 (86.0)	A: 40; B: 10	1: 38; 2: 12	BCLC B: 9; C: 41	PVTT Yes: 34; No: 16	NR^a^
Liu 2025 (24)	HAIC + targeted therapy + ICIs	50	56 (50-61)	43 (86.0)	A: 40; B: 10	1: 39; 2: 11	BCLC B: 11; C: 39	PVTT Yes: 34; No: 16	NR^a^
Ni 2026, JHC (25)	TACE-HAIC + targeted therapy + ICIs	103	53.42 ± 10.85	92 (89.3)	A: 93; B: 10	0: 96; 1: 7	BCLC B: 103; C: 0	NR	NR
Ni 2026, JHC (25)	TACE + targeted therapy + ICIs	118	53.23 ± 10.49	101 (85.6)	A: 109; B: 9	0: 113; 1: 5	BCLC B: 118; C: 0	NR	NR
Wan 2025 (26)	TACE-HAIC + targeted therapy + ICIs	35	<60: 22; ≥60: 13	29 (82.9)	A: 29; B: 6	0: 28; 1: 7	BCLC B: 4; C: 31	PVTT Yes: 25; No: 10	Yes: 12; No: 23
Wan 2025 (26)	TACE + targeted therapy + ICIs	35	<60: 22; ≥60: 13	31 (88.6)	A: 29; B: 6	0: 28; 1: 7	BCLC B: 6; C: 29	PVTT Yes: 22; No: 13	Yes: 11; No: 24
Wu 2025 (27)	TACE-HAIC + targeted therapy + ICIs	87	<60: 59; ≥60: 28	79 (90.8)	A: 74; B: 13	NR^c^	BCLC A/B: 4; C: 83	Vp3-4: 77; Others: 10	Yes: 34; No: 53
Wu 2025 (27)	HAIC + targeted therapy + ICIs	143	<60: 108; ≥60: 35	132 (92.3)	A: 126; B: 17	NR^c^	BCLC A/B: 3; C: 140	Vp3-4: 133; Others: 10	Yes: 63; No: 80
Zhou 2026 (28)	TACE-HAIC + targeted therapy + ICIs	31	≥60: 8; <60: 23	26 (83.9)	A: 28; B: 3	0: 18; 1: 13	BCLC A/B: 11; C: 20	Portal vein invasion Yes: 15; No: 16	Distant metastasis Yes: 5; No: 26
Zhou 2026 (28)	HAIC + targeted therapy + ICIs	62	≥60: 18; <60: 44	55 (88.7)	A: 56; B: 6	0: 41; 1: 21	BCLC A/B: 16; C: 46	Portal vein invasion Yes: 38; No: 24	Distant metastasis Yes: 11; No: 51
Ma 2026 (29)	TACE-HAIC + targeted therapy + ICIs	58	≤60: 33; >60: 25	48 (82.8)	A: 30; B: 28	0: 48; 1: 10	BCLC B: 35; C: 23	PVTT Yes: 21; No: 37	Yes: 12; No: 46
Ma 2026 (29)	TACE + targeted therapy + ICIs	64	≤60: 33; >60: 31	54 (84.4)	A: 31; B: 33	0: 56; 1: 8	BCLC B: 37; C: 27	PVTT Yes: 24; No: 40	Yes: 15; No: 49
Yang 2026 (30)	TACE-HAIC + targeted therapy + ICIs	35	<65: 28; ≥65: 7	34 (97.1)	A: 17; B: 18	0: 13; 1: 22	NR	PVTT Yes: 35; No: 0	Yes: 1; No: 34
Yang 2026 (30)	TACE + targeted therapy + ICIs	35	<65: 29; ≥65: 6	33 (94.3)	A: 21; B: 14	0: 15; 1: 20	NR	PVTT Yes: 35; No: 0	Yes: 1; No: 34
Hu 2026 (31)	TACE-HAIC + targeted therapy + ICIs	48	56.5 ± 9.8	41 (85.4)	A: 40; B: 8	0: 30; 1: 18	CNLC IIb: 15; IIIa: 23; IIIb: 10	Vascular invasion Yes: 29; No: 19	NR
Hu 2026 (31)	TACE + targeted therapy + ICIs	54	57.2 ± 10.3	45 (83.3)	A: 44; B: 10	0: 35; 1: 19	CNLC IIb: 18; IIIa: 26; IIIb: 10	Vascular invasion Yes: 34; No: 20	NR
Hou 2026 (32)	TACE-HAIC + targeted therapy + ICIs	79	55.1 ± 9.87	71 (89.8)	A: 61; B: 18	0-1: 50; 2: 29	BCLC C: 79	PVTT Vp2-3: 50; Vp4: 29	Yes: 15; No: 64
Hou 2026 (32)	TACE + targeted therapy + ICIs	90	55.2 ± 10.5	77 (85.5)	A: 77; B: 13	0-1: 46; 2: 44	BCLC C: 90	PVTT Vp2-3: 48; Vp4: 42	Yes: 19; No: 71
Ni 2026, BMC Cancer (33)	TACE-HAIC + targeted therapy + ICIs	51	<60: 34; ≥60: 17	43 (84.3)	A: 32; B: 19	0-1: 41; 2: 10	NR	IVC/RA TT Yes: 51; No: 0; PVTT Yes: 23; No: 28	Yes: 20; No: 31
Ni 2026, BMC Cancer (33)	HAIC + targeted therapy + ICIs	62	<60: 48; ≥60: 14	49 (79.1)	A: 37; B: 25	0-1: 44; 2: 18	NR	IVC/RA TT Yes: 62; No: 0; PVTT Yes: 19; No: 43	Yes: 15; No: 47

Baseline characteristics are presented as reported in the original studies. Post-PSM values are shown when available for matched cohorts, whereas original observed cohorts are presented for IPTW studies. BCLC and CNLC categories were not reclassified, and definitions of PVTT or other vascular invasion were retained as originally reported. a Liu 2025 reported inconsistent post-PSM extrahepatic metastasis counts; therefore, this variable was recorded as NR. b For Li 2025, treatment-group assignment was based on the abstract, Results section, and outcome denominators because the baseline table appeared to be inconsistent with the reported treatment groups. c Wu 2025 reported ECOG PS 0–2 as an eligibility criterion but did not provide baseline category distribution. NR, not reported; ECOG PS, Eastern Cooperative Oncology Group performance status; BCLC, Barcelona Clinic Liver Cancer; CNLC, China Liver Cancer; PVTT, portal vein tumor thrombosis; IVC/RA TT, inferior vena cava/right atrial tumor thrombus; ICIs, immune checkpoint inhibitors; PSM, propensity score matching; IPTW, inverse probability of treatment weighting.

**Table 1b:** Study design, treatment regimens, and analysis populations.

Study	Comparator	Study design/adjustment	TACE-HAIC regimen	Control regimen	Analysis population
Chi 2025 (20)	TACE-based	Retrospective cohort; unadjusted	TACE + HAIC + lenvatinib + anti-PD-1 antibody	TACE + lenvatinib + anti-PD-1 antibody	Original: 24 vs 38; analysis: 24 vs 38
Chi 2025 (20)	HAIC-based	Retrospective cohort; unadjusted	TACE + HAIC + lenvatinib + anti-PD-1 antibody	HAIC + lenvatinib + anti-PD-1 antibody	Original: 24 vs 12; analysis: 24 vs 12
Huang 2024 (21)	TACE-based	Retrospective cohort; multivariable Cox	TACE + HAIC + TKIs + PD-1 inhibitors	TACE + TKIs + PD-1 inhibitors	Original: 63 vs 60; analysis: 63 vs 60
Feng 2026 (22)	TACE-based	Retrospective cohort; 1:1 PSM	TACE + FOLFOX/RALOX-HAIC + targeted therapy + ICIs	TACE + targeted therapy + ICIs	Original: 156 vs 118; efficacy/survival: 107 vs 107 (PSM); safety: 156 vs 118
Li 2025 (23)	TACE-based	Retrospective cohort; IPTW	cTACE/DEB-TACE + FOLFOX/RALOX-HAIC + targeted therapy + ICIs	cTACE/DEB-TACE + targeted therapy + ICIs	Original: 154 vs 305; survival: IPTW; response/safety: 154 vs 305
Liu 2025 (24)	HAIC-based	Retrospective cohort; 1:1 PSM	TACE + HAIC + donafenib/lenvatinib + ICIs	HAIC + donafenib/lenvatinib + ICIs	Original: 103 vs 94; efficacy/survival/safety: 50 vs 50 (PSM)
Ni 2026, JHC (25)	TACE-based	Multicenter retrospective cohort; unadjusted	cTACE + HAIC + lenvatinib + PD-1 inhibitors	cTACE + lenvatinib + PD-1 inhibitors	Original: 103 vs 118; analysis: 103 vs 118
Wan 2025 (26)	TACE-based	Retrospective quality-improvement study; 1:1 PSM	TACE + HAIC + targeted therapy + ICIs	TACE + targeted therapy + ICIs	Original: 102 vs 56; efficacy/survival: 35 vs 35 (PSM); safety: 102 vs 56
Wu 2025 (27)	HAIC-based	Retrospective cohort; PSM	TACE + HAIC + TKIs + ICIs	HAIC + TKIs + ICIs	Original: 119 vs 244; efficacy/survival/safety: 87 vs 143 (PSM)
Zhou 2026 (28)	HAIC-based	Multicenter retrospective cohort; 1:2 PSM	cTACE/DEB-TACE + HAIC + targeted therapy + ICIs	HAIC + targeted therapy + ICIs	Original: 41 vs 172; efficacy/survival: 31 vs 62 (PSM); safety: 31 vs 61
Ma 2026 (29)	TACE-based	Multicenter retrospective cohort; IPTW	DEB-TACE + HAIC + donafenib + camrelizumab	DEB-TACE + donafenib + camrelizumab	Original: 58 vs 64; survival: IPTW; response/safety: 58 vs 64
Yang 2026 (30)	TACE-based	Retrospective cohort; 1:1 PSM	TACE + HAIC + lenvatinib + camrelizumab/sintilimab	TACE + lenvatinib + camrelizumab/sintilimab	Original: 42 vs 72; efficacy/survival/safety: 35 vs 35 (PSM)
Hu 2026 (31)	TACE-based	Retrospective cohort; unadjusted	TACE + HAIC + lenvatinib + camrelizumab	TACE + lenvatinib + camrelizumab	Original: 48 vs 54; response/safety: 48 vs 54; survival: not pooled
Hou 2026 (32)	TACE-based	Retrospective cohort; unadjusted	TACE + FOLFOX-HAIC + lenvatinib/donafenib + camrelizumab/sintilimab	TACE + lenvatinib/donafenib + camrelizumab/sintilimab	Original: 79 vs 90; analysis: 79 vs 90
Ni 2026, BMC Cancer (33)	HAIC-based	Multicenter retrospective cohort; unadjusted	dTACE + HAIC + targeted therapy + PD-1 inhibitors	HAIC + targeted therapy + PD-1 inhibitors	Original: 51 vs 62; analysis: 51 vs 62

Comparator indicates the control group receiving a single transarterial modality in the corresponding analysis. Regimens are summarized using standardized terminology, while specific TACE/HAIC techniques and agents are retained where reported. For IPTW studies, original enrolled patient numbers are presented, and weighted pseudo-populations are not considered additional patients. PSM, propensity score matching; IPTW, inverse probability of treatment weighting; cTACE, conventional TACE; DEB-TACE, drug-eluting bead TACE; FOLFOX, oxaliplatin, fluorouracil, and leucovorin; RALOX, raltitrexed and oxaliplatin; TKIs, tyrosine kinase inhibitors; ICIs, immune checkpoint inhibitors.

### Risk of bias and certainty of evidence

NOS scores ranged from 6 to 8 across the included studies. For the survival outcomes, the ROBINS-I assessment indicated a moderate-to-serious overall risk of bias, mainly related to confounding and participant selection. No included survival comparison was judged to be at low overall risk of bias. GRADE certainty ranged from low to very low across outcomes. Detailed NOS, ROBINS-I, and GRADE assessments are provided in **Supplementary Tables S2**–**S4**.

### TACE-based comparison

#### Overall survival

Nine studies contributed to the TACE-based OS analysis. TACE-HAIC plus targeted therapy and ICIs was associated with a significantly lower hazard of death than TACE plus targeted therapy and ICIs (HR = 0.59, 95% CI: 0.47–0.75; I² = 54.7%) (**Figure 2**).

**Figure 2:**
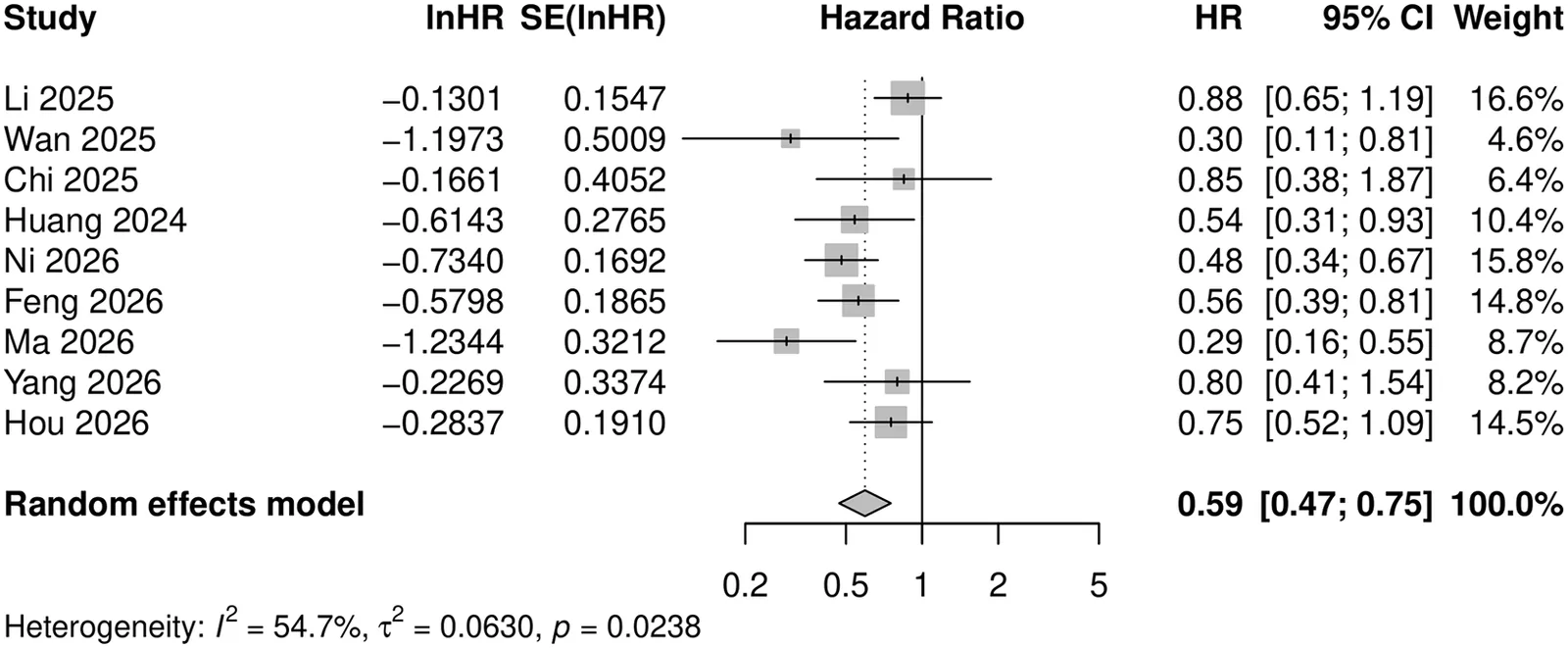
Forest plot of overall survival in the TACE-based comparison.

#### Progression-free survival

Eight studies contributed to the PFS analysis. TACE-HAIC plus targeted therapy and ICIs was associated with a significantly lower hazard of progression or death than TACE plus targeted therapy and ICIs (HR = 0.65, 95% CI: 0.55–0.78; I² = 36.2%) (**Figure 3**).

**Figure 3:**
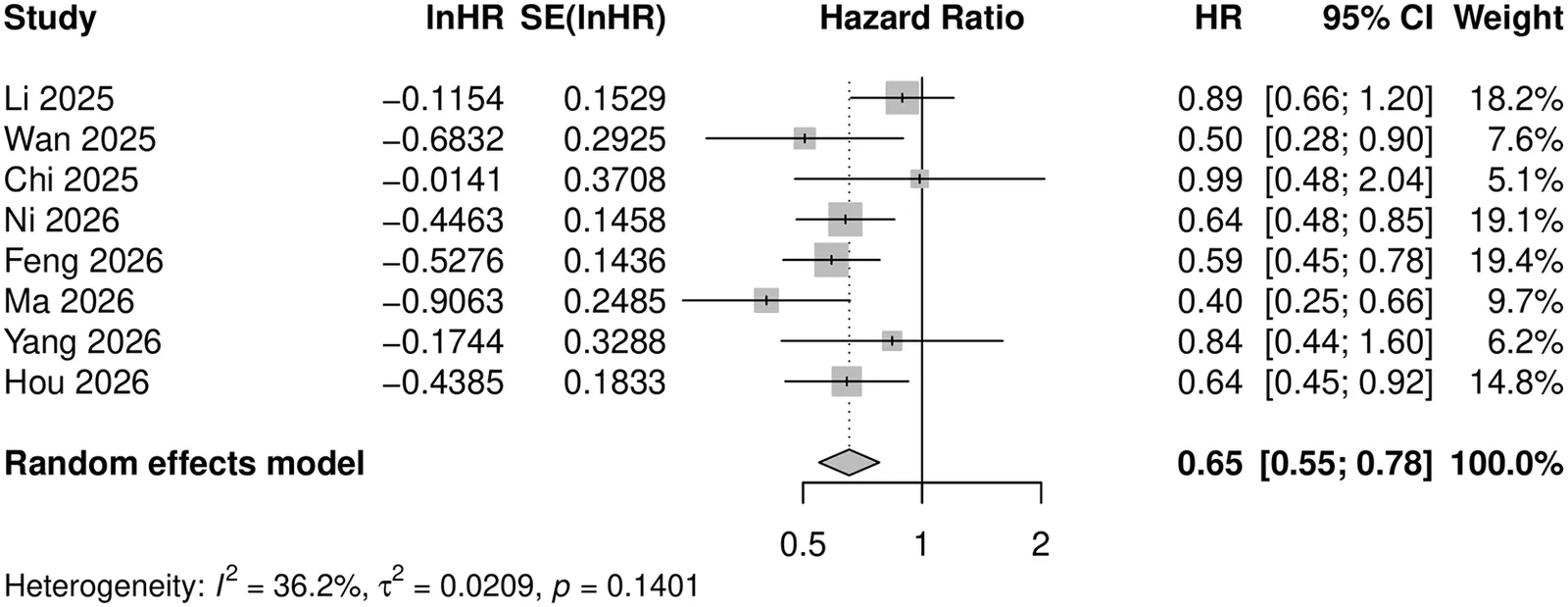
Forest plot of progression-free survival in the TACE-based comparison.

#### Objective response rate

Nine studies reported ORR. TACE-HAIC plus targeted therapy and ICIs was associated with a significantly higher ORR than the TACE-based comparator (RR = 1.34, 95% CI: 1.19–1.52; I² = 0.3%) (**Figure 4**).

**Figure 4:**
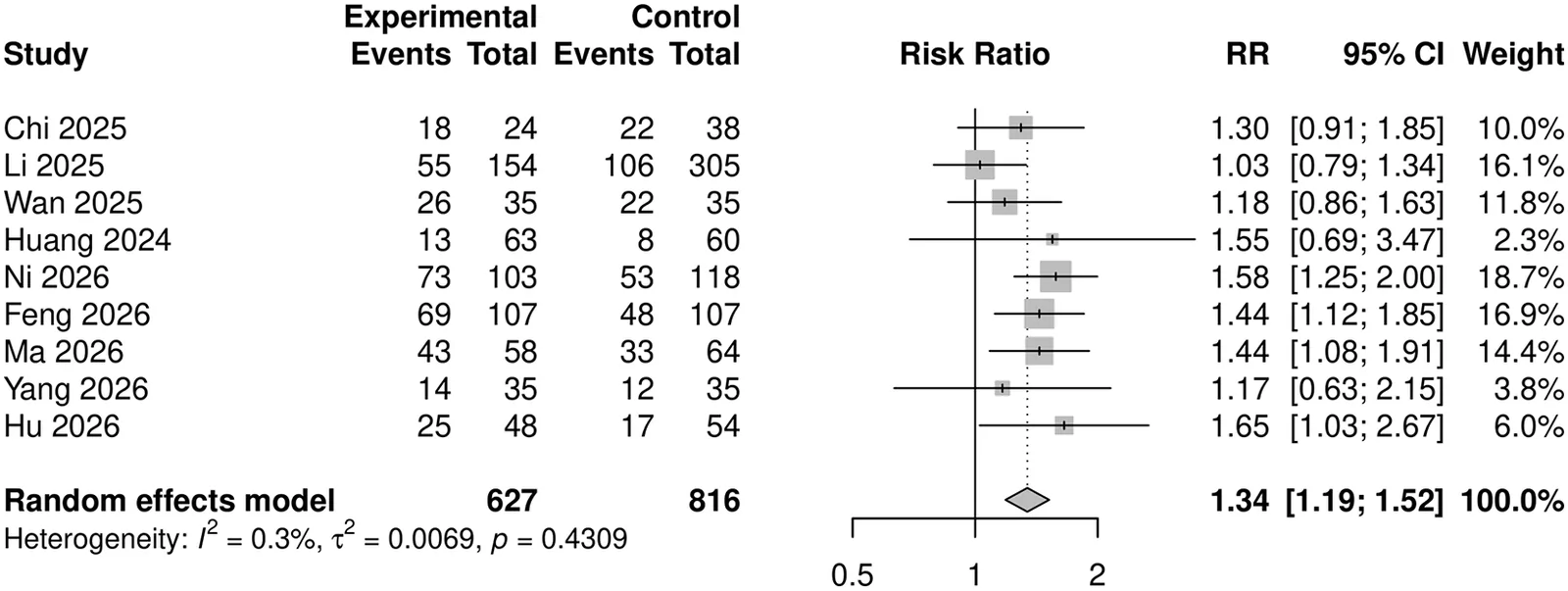
Forest plot of objective response rate in the TACE-based comparison.

#### Disease control rate

Nine studies reported DCR. TACE-HAIC plus targeted therapy and ICIs was associated with a significantly higher DCR than the TACE-based comparator (RR = 1.14, 95% CI: 1.07–1.21; I² = 50.5%) (**Figure 5**).

**Figure 5:**
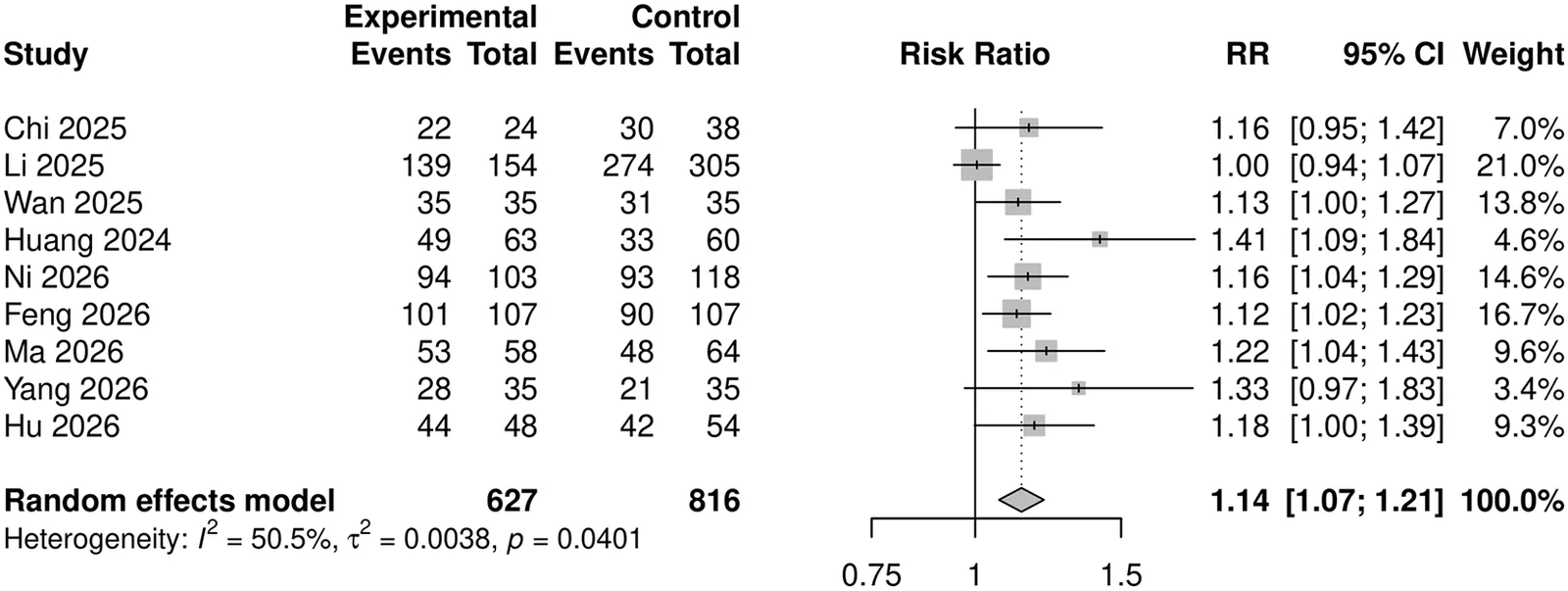
Forest plot of disease control rate in the TACE-based comparison.

#### Complete response

Nine studies reported CR. TACE-HAIC plus targeted therapy and ICIs was associated with a significantly higher CR rate than the TACE-based comparator (RR = 2.17, 95% CI: 1.46–3.21; I² = 0%) (**Figure 6**).

**Figure 6:**
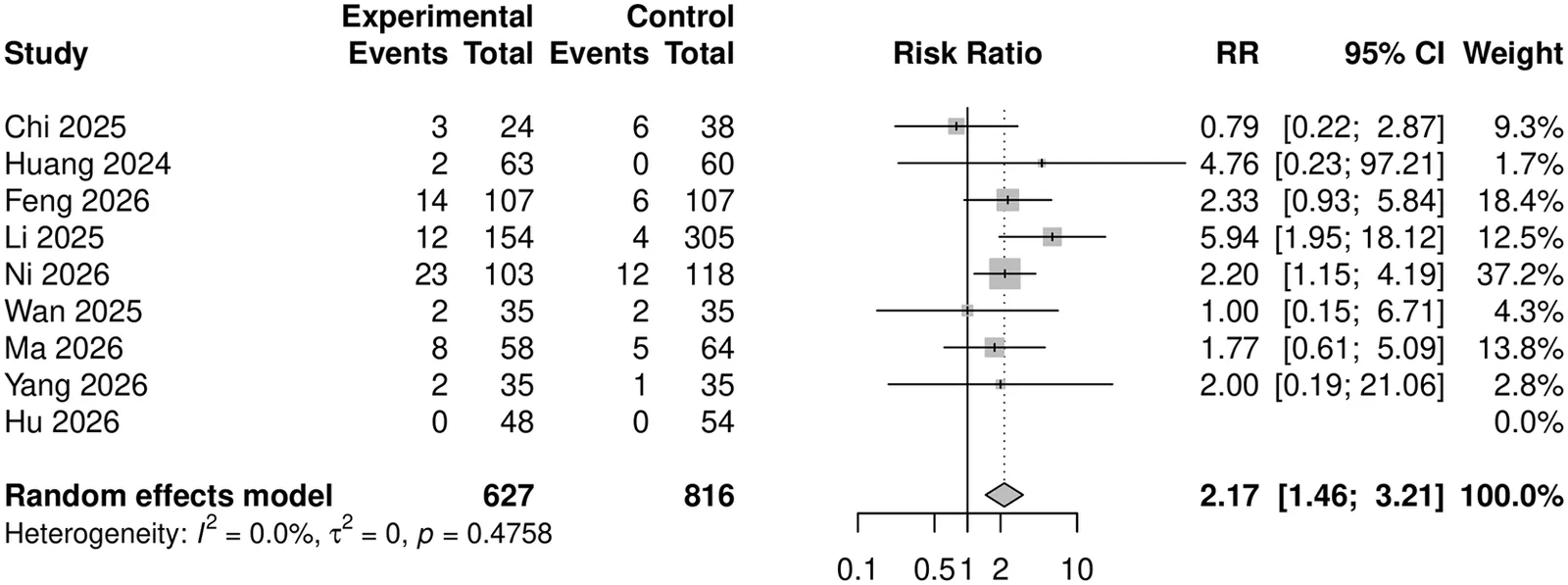
Forest plot of complete response in the TACE-based comparison.

### HAIC-based comparison

#### Overall survival

Five studies contributed to the HAIC-based OS analysis. TACE-HAIC plus targeted therapy and ICIs was associated with a significantly lower hazard of death than HAIC plus targeted therapy and ICIs (HR = 0.56, 95% CI: 0.43–0.72; I² = 0%) (**Figure 7**).

**Figure 7:**
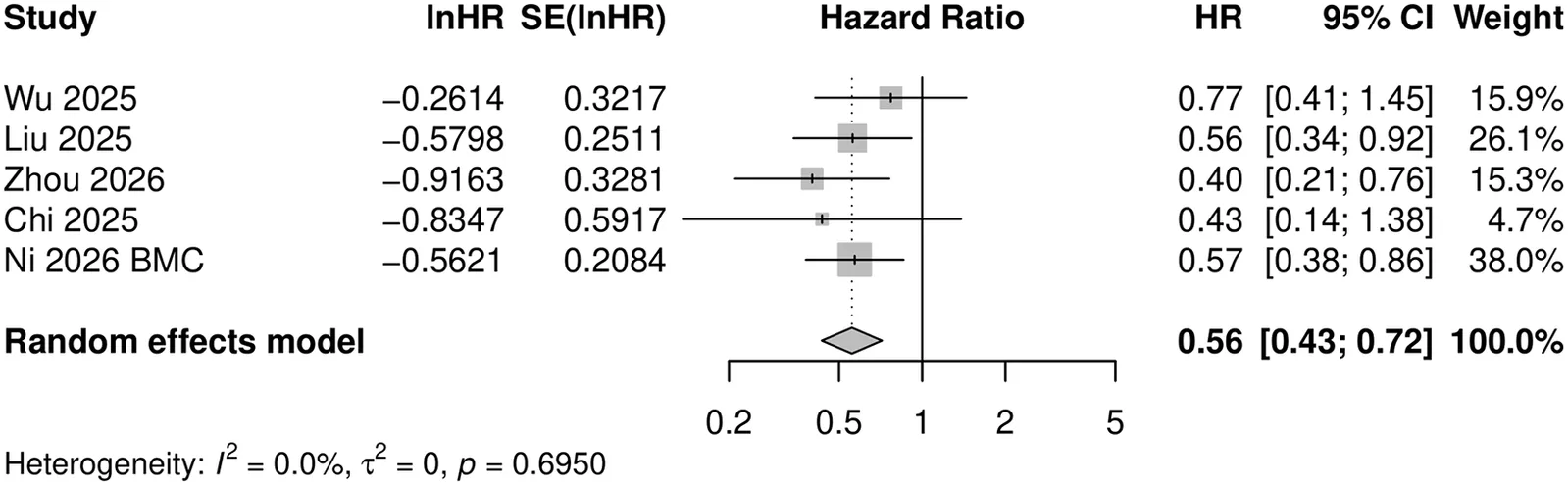
Forest plot of overall survival in the HAIC-based comparison.

#### Progression-free survival

Four studies contributed to the PFS analysis. TACE-HAIC plus targeted therapy and ICIs was associated with a significantly lower hazard of progression or death than HAIC plus targeted therapy and ICIs (HR = 0.64, 95% CI: 0.48–0.86; I² = 42.3%) (**Figure 8**).

**Figure 8:**
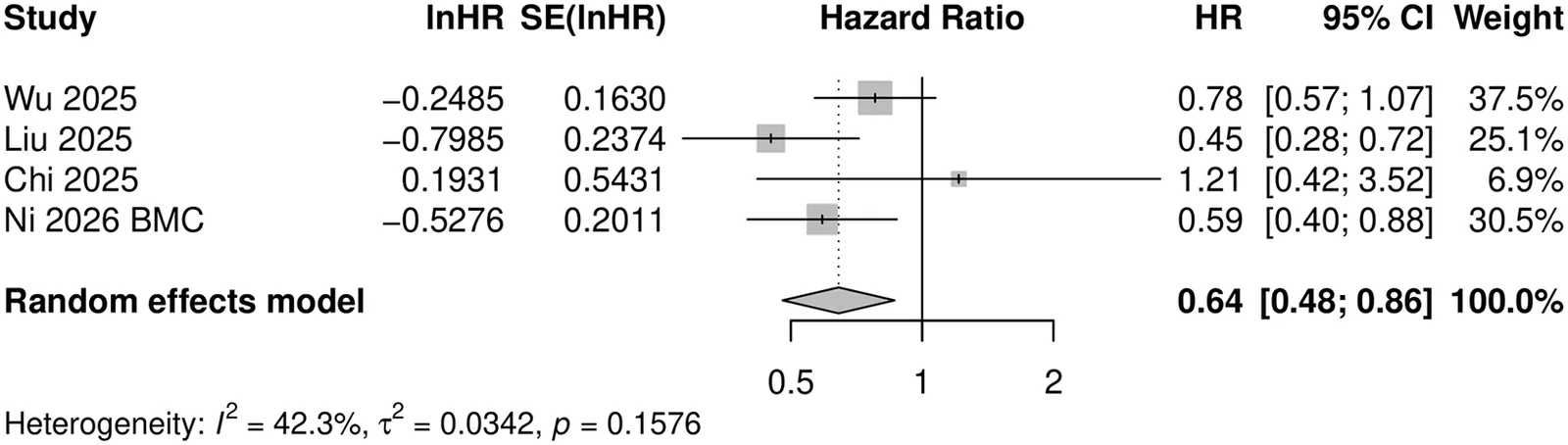
Forest plot of progression-free survival in the HAIC-based comparison.

#### Objective response rate

Five studies reported ORR. TACE-HAIC plus targeted therapy and ICIs was associated with a significantly higher ORR than the HAIC-based comparator (RR = 1.24, 95% CI: 1.03–1.50; I² = 27.5%) (**Figure 9**).

**Figure 9:**
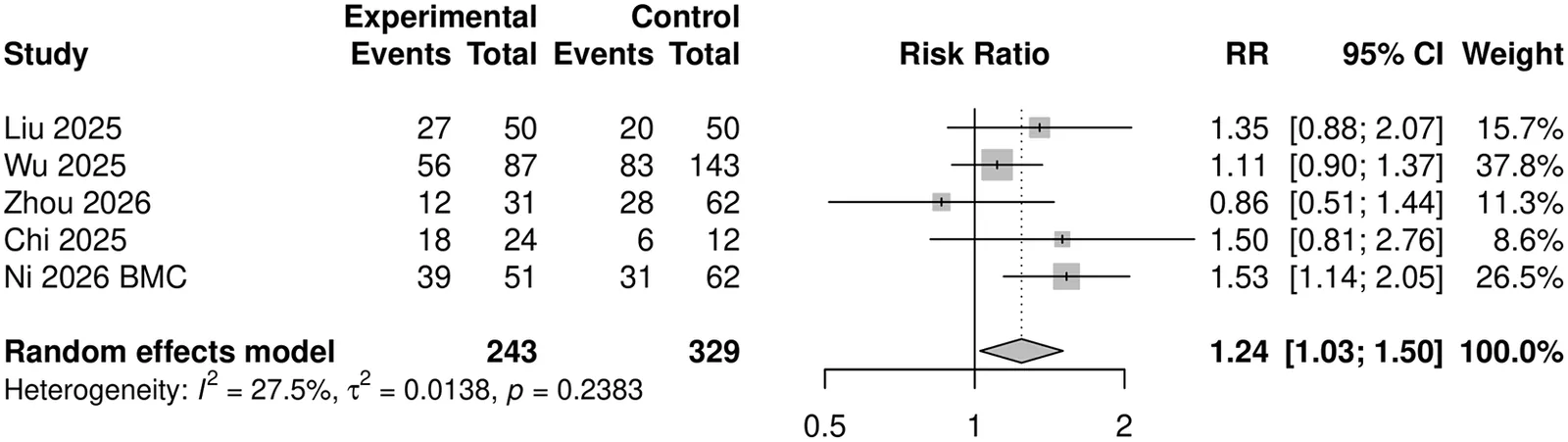
Forest plot of objective response rate in the HAIC-based comparison.

#### Disease control rate

Five studies reported DCR. The pooled estimate did not show a clear between-group difference (RR = 1.09, 95% CI: 0.99–1.19; I² = 33.4%) (**Figure 10**).

**Figure 10:**
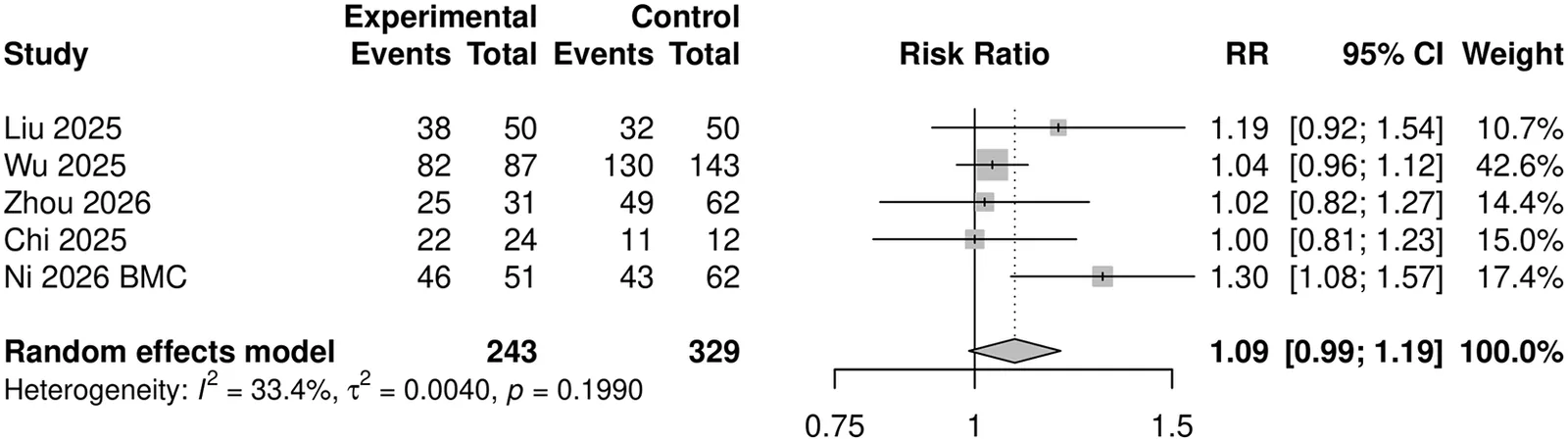
Forest plot of disease control rate in the HAIC-based comparison.

#### Complete response

Five studies reported CR. The pooled estimate was imprecise and did not demonstrate a clear between-group difference (RR = 1.62, 95% CI: 0.61–4.30; I² = 0%) (**Figure 11**).

**Figure 11:**
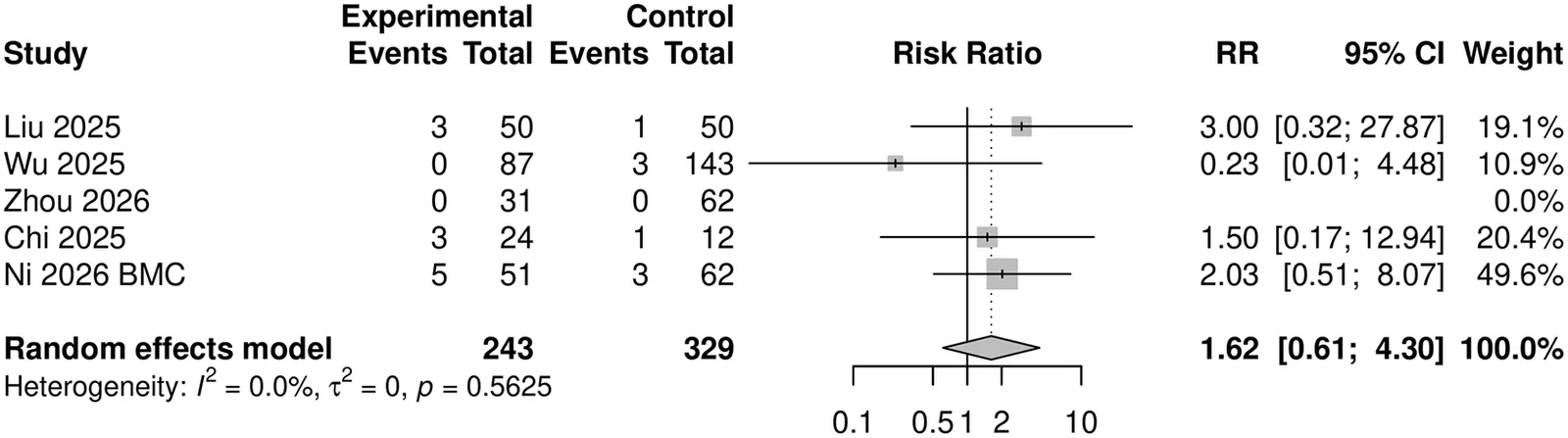
Forest plot of complete response in the HAIC-based comparison.

### Safety analysis

For overall grade ≥3 TRAEs, the TACE-based comparison included four reporting studies, of which three were informative (43/175 vs. 45/210 events; RR = 1.16, 95% CI: 0.81–1.67; I² = 0%). The HAIC-based comparison included three informative studies (51/125 vs. 41/124 events; RR = 1.27, 95% CI: 0.92–1.74; I² = 0%). The pooled estimates were imprecise in both comparisons, with confidence intervals that did not exclude clinically important harm. Therefore, the available evidence was insufficient to determine whether TACE-HAIC increases the risk of overall grade ≥3 TRAEs (**Figure 12**).

**Figure 12:**
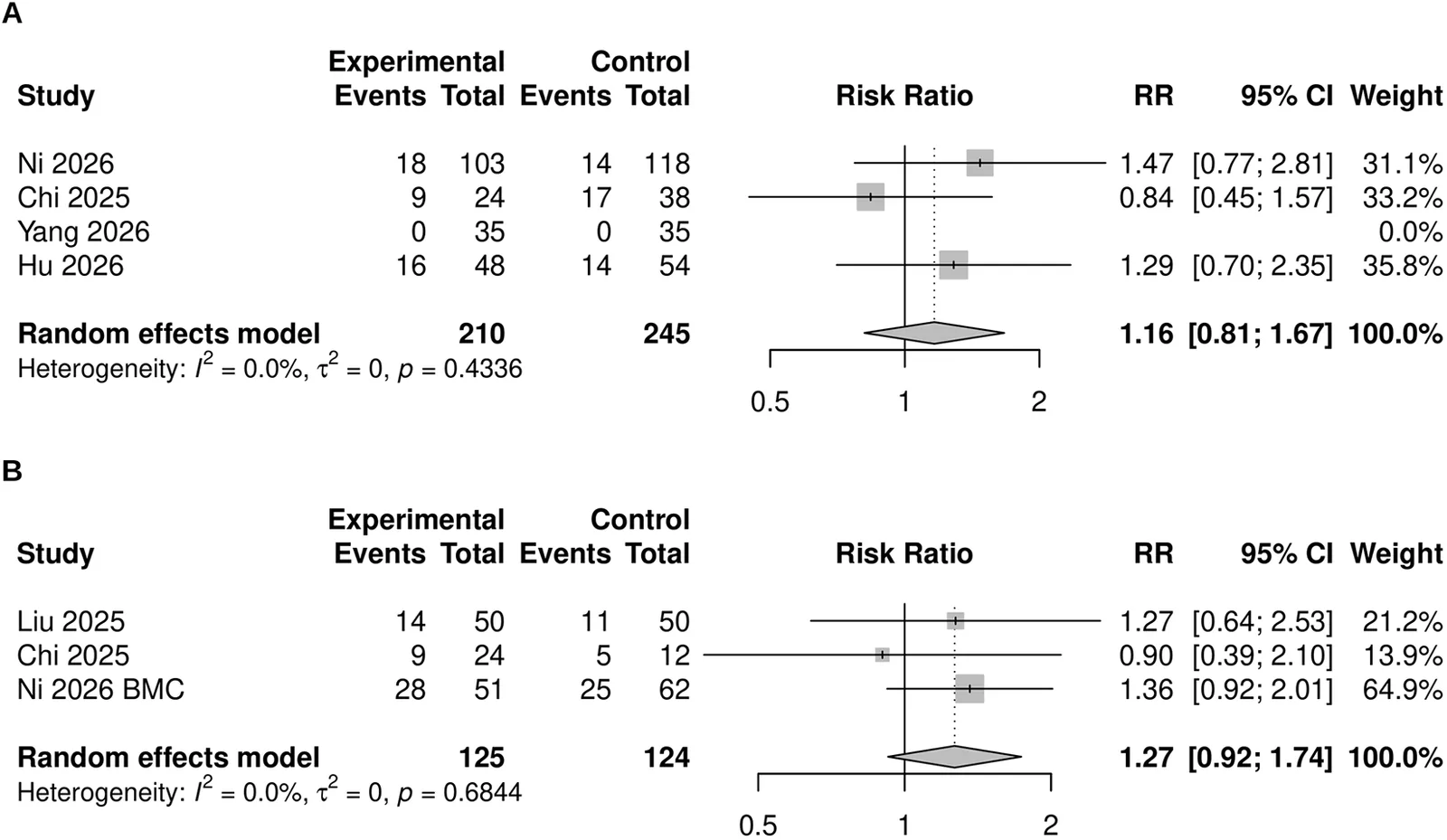
Forest plots of overall grade ≥3 treatment-related adverse events. **(A)** TACE-based comparison; **(B)** HAIC-based comparison.

Exploratory individual-AE analyses showed that, in the TACE-based comparison, TACE-HAIC plus targeted therapy and ICIs was associated with higher risks of any-grade hypoalbuminemia, nausea, vomiting, and decreased appetite. In the HAIC-based comparison, higher risks were observed for any-grade ALT elevation, hypoalbuminemia, fever, and abdominal pain, and for grade ≥3 ALT elevation, AST elevation, and abdominal pain. Detailed AE results are provided in **Supplementary Tables S6a, b**, and **S7**.

### Sensitivity analysis

After excluding studies at serious overall risk of bias according to ROBINS-I, the pooled effects in the TACE-based comparison remained significant for OS (HR = 0.45, 95% CI: 0.32–0.63; I² = 27.6%) and PFS (HR = 0.53, 95% CI: 0.41–0.67; I² = 0%). In the HAIC-based comparison, OS remained significant (HR = 0.56, 95% CI: 0.40–0.78; I² = 1.5%), whereas PFS no longer reached statistical significance (HR = 0.61, 95% CI: 0.36–1.04; I² = 72.6%).

Leave-one-out analyses showed that all TACE-based efficacy outcomes remained statistically significant after the omission of any single study. In the HAIC-based comparison, OS remained significant throughout, whereas PFS lost statistical significance after the omission of Ni 2026 (BMC Cancer), and ORR was no longer statistically significant after the omission of Liu 2025, Chi 2025, or Ni 2026 (BMC Cancer); DCR and CR remained non-significant.

Given its substantial weight and comparatively attenuated effect estimates, the influence of Li 2025 was examined separately. Excluding Li 2025 did not materially alter the TACE-based findings, with pooled estimates of HR = 0.55 (95% CI: 0.45–0.69; I² = 36.7%) for OS and HR = 0.61 (95% CI: 0.52–0.71; I² = 0.7%) for PFS. The corresponding pooled RRs were 1.42 (95% CI: 1.26–1.60; I² = 0%) for ORR, 1.16 (95% CI: 1.10–1.22; I² = 0%) for DCR, and 1.88 (95% CI: 1.23–2.86; I² = 0%) for CR. Detailed sensitivity analyses are presented in **Supplementary Table S8** and **Supplementary Figures S1**–**S4**.

## Discussion

This meta-analysis separately evaluated TACE-based and HAIC-based comparisons of TACE-HAIC plus targeted therapy and ICIs for unresectable HCC. Compared with TACE plus targeted therapy and ICIs, TACE-HAIC was associated with a lower hazard of death, with consistent supportive findings across PFS and tumor-response outcomes. Compared with HAIC plus targeted therapy and ICIs, TACE-HAIC was also associated with a lower hazard of death; PFS and ORR favored TACE-HAIC in the main analyses, although these secondary findings were less robust in sensitivity analyses. Available evidence was insufficient to determine whether TACE-HAIC increases the overall risk of grade ≥3 TRAEs.

HCC features an insidious onset, rapid intrahepatic progression, and frequent vascular invasion (2, 3). Patients with intermediate-to-advanced-stage uHCC commonly present with HTB, PVTT, and multifocal intrahepatic disease. Under these circumstances, a single locoregional modality combined with targeted therapy and ICIs may provide insufficient intrahepatic tumor control in some patients, whereas combining TACE and HAIC may offer complementary locoregional effects. TACE induces hypoxia and ischemia by embolizing tumor-feeding arteries, thereby reducing tumor perfusion and prolonging local retention of chemotherapeutic agents (4). HAIC, in turn, continuously infuses chemotherapeutic agents through the hepatic artery, maintaining elevated drug levels within the local tumor microenvironment and potentially targeting residual viable lesions and intrahepatic micrometastases (39). TACE relies primarily on embolization with locally delivered chemotherapy, whereas HAIC generally employs continuous hepatic arterial infusion of regimens such as FOLFOX. Differences in chemotherapeutic composition and drug delivery between the two approaches may provide complementary antitumor effects (40). The hypoxic microenvironment after TACE activates the HIF-1α/VEGF signaling axis, promoting aberrant angiogenesis, collateral vessel formation, and immunosuppression (41). HAIC may enhance tumor-cell injury, antigen release, and immunogenic cell death through sustained regional chemotherapy (42). TKIs or anti-VEGF/VEGFR agents suppress VEGF/VEGFR, FGF/FGFR, and PDGFR signaling and may promote vascular normalization and immune-cell infiltration (43, 44), whereas PD-1/PD-L1 inhibitors restore antitumor T-cell responses (45). These complementary mechanisms provide a biologic rationale for the combination, although they do not establish causality for the observed clinical associations.

The more consistent TACE-based findings may reflect a broader incremental contribution from adding HAIC. Huang et al. observed a greater improvement in DCR than in ORR, suggesting that the benefit may involve disease stabilization rather than tumor shrinkage alone (21). In contrast, the large IPTW study by Li et al. showed a more attenuated effect in a clinically heterogeneous population, indicating that the magnitude of benefit may depend on patient selection and treatment implementation (23). The HAIC-based setting appeared more context dependent: Wu et al. reported substantial tumor control with the HAIC-based regimen alone in patients with HTB and Vp3–4 PVTT, whereas Ni et al. observed a more favorable effect of adding TACE in a cohort with extensive macrovascular invasion (27, 33). These findings suggest that the incremental effect of adding HAIC to TACE may be more consistent, whereas the incremental effect of adding TACE to HAIC may depend more strongly on baseline disease characteristics and the effectiveness of the underlying HAIC-based regimen.

The higher CR rate observed in the TACE-based comparison may indicate a greater depth of radiologic response and could be relevant to conversion strategies in HCC. Zhao et al. reported favorable conversion outcomes with TACE-HAIC plus a PD-1 inhibitor and lenvatinib (46); however, conversion resection was not a pooled endpoint in the present meta-analysis, and radiologic CR does not itself establish resectability.

Moderate heterogeneity was observed in several TACE-based outcomes. Li (2025) was potentially influential because of its relatively large sample size and comparatively attenuated effect estimates. Excluding this study reduced heterogeneity in OS (I²: 54.7% to 36.7%), PFS (36.2% to 0.7%), and DCR (50.5% to 0%), while the pooled efficacy estimates remained statistically significant. Thus, Li (2025) appeared to contribute to between-study heterogeneity but did not account for the overall treatment association. Residual heterogeneity may reflect differences in patient populations, treatment protocols, follow-up, and analytic methods, although these factors could not be formally examined by meta-regression because of the limited number of studies.

Regarding safety, the pooled estimates for overall grade ≥3 TRAEs were imprecise in both the TACE-based and HAIC-based comparisons, with confidence intervals that did not exclude clinically important harm, and the certainty of evidence was very low. Exploratory analyses suggested higher risks of several gastrointestinal and hepatic adverse events, but these findings should be interpreted cautiously because of heterogeneous reporting and sparse events. Longer-term hepatic deterioration, treatment discontinuation, and cumulative toxicity were insufficiently reported.

There are several limitations. First, all included studies were retrospective cohorts, and residual confounding cannot be excluded. Confounding adjustment varied across studies and outcomes; although many survival estimates were propensity-adjusted or multivariable-adjusted, several survival, tumor-response, and safety comparisons were based on unadjusted treatment estimates or raw event counts. This limitation is particularly relevant because no included survival comparison was judged to be at low overall risk of bias by ROBINS-I. Second, all studies were conducted in China, where HBV-related HCC and the use of HAIC are relatively common (47, 48), which may limit the generalizability of the findings to other populations and treatment settings. Third, substantial clinical and methodological variation existed in tumor burden, vascular invasion, extrahepatic metastasis, locoregional and systemic treatment regimens, response assessment, follow-up, and analytic methods. Post-progression treatment and conversion surgery were also incompletely reported. Fourth, the number of studies within each comparator-specific analysis was insufficient for reliable meta-regression or formal assessment of small-study effects, precluding further exploration of potential effect modifiers such as tumor stage, vascular invasion, and specific treatment regimens. Finally, safety data were sparse and inconsistently reported, particularly for rare and long-term toxicities. These limitations should be considered when interpreting the magnitude and generalizability of the observed associations.

## Conclusion

TACE-HAIC plus targeted therapy and ICIs was associated with a lower hazard of death compared with both TACE plus targeted therapy and ICIs and HAIC plus targeted therapy and ICIs in patients with uHCC. In the TACE-based comparison, this survival association was accompanied by consistently favorable findings across secondary efficacy outcomes, whereas the evidence from secondary outcomes was less consistent in the HAIC-based comparison. Available evidence was insufficient to determine whether TACE-HAIC increases the overall risk of grade ≥3 TRAEs. Given the retrospective evidence base and the low or very low certainty of the evidence, prospective comparative studies are needed to validate these findings, identify patients most likely to benefit, and better characterize long-term safety.

## Data Availability

The original contributions presented in the study are included in the article/**Supplementary Material**. Further inquiries can be directed to the corresponding author.
