# Supplementary material for: TACE-HAIC plus targeted therapy and immune checkpoint inhibitors for unresectable hepatocellular carcinoma: a systematic review and meta-analysis of TACE- and HAIC-based comparisons
Source: Front Oncol. 2026 Oct 23;16:1942009. doi: 10.3389/fonc.2026.1942009 (PMC13739493; doi:10.3389/fonc.2026.1942009)
Supplement: Supplementary file 1 [file Supplementaryfile1.docx]

| **Section and Topic** | **Item #** | **Checklist item** | **Location where item is reported** |
| --- | --- | --- | --- |
| **TITLE** | | |  |
| Title | 1 | Identify the report as a systematic review. | Main manuscript: Title. |
| **ABSTRACT** | | |  |
| Abstract | 2 | See the PRISMA 2020 for Abstracts checklist. | Main manuscript: Abstract. |
| **INTRODUCTION** | | |  |
| Rationale | 3 | Describe the rationale for the review in the context of existing knowledge. | Main manuscript: Introduction. |
| Objectives | 4 | Provide an explicit statement of the objective(s) or question(s) the review addresses. | Main manuscript: Introduction (final paragraph stating the review objective). |
| **METHODS** | | |  |
| Eligibility criteria | 5 | Specify the inclusion and exclusion criteria for the review and how studies were grouped for the syntheses. | Main manuscript: Methods - Eligibility Criteria; Methods - Outcomes and Statistical Analysis; Tables 1a-1b. |
| Information sources | 6 | Specify all databases, registers, websites, organisations, reference lists and other sources searched or consulted to identify studies. Specify the date when each source was last searched or consulted. | Main manuscript: Methods - Literature Search (last search: Aug 19, 2026); Supplementary Table S1. |
| Search strategy | 7 | Present the full search strategies for all databases, registers and websites, including any filters and limits used. | Supplementary Table S1 (complete database-specific search strategies; only final combined hit counts are reported). |
| Selection process | 8 | Specify the methods used to decide whether a study met the inclusion criteria of the review, including how many reviewers screened each record and each report retrieved, whether they worked independently, and if applicable, details of automation tools used in the process. | Main manuscript: Methods - Data Extraction and Risk-of-Bias Assessment (independent screening by two investigators and disagreement resolution). |
| Data collection process | 9 | Specify the methods used to collect data from reports, including how many reviewers collected data from each report, whether they worked independently, any processes for obtaining or confirming data from study investigators, and if applicable, details of automation tools used in the process. | Main manuscript: Methods - Data Extraction and Risk-of-Bias Assessment (independent data extraction by two investigators and disagreement resolution). |
| Data items | 10a | List and define all outcomes for which data were sought. Specify whether all results that were compatible with each outcome domain in each study were sought (e.g. for all measures, time points, analyses), and if not, the methods used to decide which results to collect. | Main manuscript: Methods - Eligibility Criteria; Methods - Outcomes and Statistical Analysis. |
|  | 10b | List and define all other variables for which data were sought (e.g. participant and intervention characteristics, funding sources). Describe any assumptions made about any missing or unclear information. | Main manuscript: Methods - Data Extraction and Risk-of-Bias Assessment; Tables 1a-1b; Supplementary Table S5. |
| Study risk of bias assessment | 11 | Specify the methods used to assess risk of bias in the included studies, including details of the tool(s) used, how many reviewers assessed each study and whether they worked independently, and if applicable, details of automation tools used in the process. | Main manuscript: Methods - Data Extraction and Risk-of-Bias Assessment; Supplementary Table S3. |
| Effect measures | 12 | Specify for each outcome the effect measure(s) (e.g. risk ratio, mean difference) used in the synthesis or presentation of results. | Main manuscript: Methods - Outcomes and Statistical Analysis. |
| Synthesis methods | 13a | Describe the processes used to decide which studies were eligible for each synthesis (e.g. tabulating the study intervention characteristics and comparing against the planned groups for each synthesis (item #5)). | Main manuscript: Methods - Eligibility Criteria; Methods - Outcomes and Statistical Analysis; Table 1b. |
|  | 13b | Describe any methods required to prepare the data for presentation or synthesis, such as handling of missing summary statistics, or data conversions. | Main manuscript: Methods - Data Extraction and Risk-of-Bias Assessment; Supplementary Table S5; Supplementary Tables S6-S7. |
|  | 13c | Describe any methods used to tabulate or visually display results of individual studies and syntheses. | Tables 1a-1b; Figures 2-12; Supplementary Tables S4-S8; Supplementary Figure legends. |
|  | 13d | Describe any methods used to synthesize results and provide a rationale for the choice(s). If meta-analysis was performed, describe the model(s), method(s) to identify the presence and extent of statistical heterogeneity, and software package(s) used. | Main manuscript: Methods - Outcomes and Statistical Analysis (random-effects models, REML, Q/I2/tau2, R 4.5.2). |
|  | 13e | Describe any methods used to explore possible causes of heterogeneity among study results (e.g. subgroup analysis, meta-regression). | Main manuscript: Methods - Outcomes and Statistical Analysis; Discussion; Supplementary Table S8. Meta-regression was not feasible (<10 studies per outcome). |
|  | 13f | Describe any sensitivity analyses conducted to assess robustness of the synthesized results. | Main manuscript: Methods - Outcomes and Statistical Analysis; Results - Sensitivity Analysis; Supplementary Table S8; Supplementary Figures S1-S4. |
| Reporting bias assessment | 14 | Describe any methods used to assess risk of bias due to missing results in a synthesis (arising from reporting biases). | Main manuscript: Methods - Outcomes and Statistical Analysis. Formal publication-bias assessment was not performed because fewer than 10 studies were available per comparator-specific outcome. |
| Certainty assessment | 15 | Describe any methods used to assess certainty (or confidence) in the body of evidence for an outcome. | Main manuscript: Methods - Data Extraction and Risk-of-Bias Assessment; Results - Risk of Bias and Certainty of Evidence; Supplementary Table S4. |
| **RESULTS** | | |  |
| Study selection | 16a | Describe the results of the search and selection process, from the number of records identified in the search to the number of studies included in the review, ideally using a flow diagram. | Main manuscript: Results - Study Selection; Figure 1 (3,612 records identified, 1,417 duplicates removed, 2,195 records screened, 101 reports assessed in detail, 87 reports excluded, and 14 studies included). |
|  | 16b | Cite studies that might appear to meet the inclusion criteria, but which were excluded, and explain why they were excluded. | Supplementary Table S9 (87 reports excluded after detailed eligibility assessment, with citation/title and primary exclusion reason). |
| Study characteristics | 17 | Cite each included study and present its characteristics. | Main manuscript: Results - Baseline Study Characteristics; Tables 1a-1b; included studies cited as References 20-33. |
| Risk of bias in studies | 18 | Present assessments of risk of bias for each included study. | Main manuscript: Results - Risk of Bias and Certainty of Evidence; Supplementary Table S3. |
| Results of individual studies | 19 | For all outcomes, present, for each study: (a) summary statistics for each group (where appropriate) and (b) an effect estimate and its precision (e.g. confidence/credible interval), ideally using structured tables or plots. | Figures 2-12; Supplementary Tables S6a-S7. |
| Results of syntheses | 20a | For each synthesis, briefly summarise the characteristics and risk of bias among contributing studies. | Main manuscript: Results - Baseline Study Characteristics; Tables 1a-1b; Results - Risk of Bias and Certainty of Evidence; Supplementary Table S3. |
|  | 20b | Present results of all statistical syntheses conducted. If meta-analysis was done, present for each the summary estimate and its precision (e.g. confidence/credible interval) and measures of statistical heterogeneity. If comparing groups, describe the direction of the effect. | Main manuscript: Results - TACE-Based Comparison, HAIC-Based Comparison, and Safety Analysis; Figures 2-12; Supplementary Tables S6a-S7. |
|  | 20c | Present results of all investigations of possible causes of heterogeneity among study results. | Main manuscript: Discussion; Supplementary Table S8. No formal meta-regression was performed. |
|  | 20d | Present results of all sensitivity analyses conducted to assess the robustness of the synthesized results. | Main manuscript: Results - Sensitivity Analysis; Supplementary Table S8; Supplementary Figures S1-S4. |
| Reporting biases | 21 | Present assessments of risk of bias due to missing results (arising from reporting biases) for each synthesis assessed. | Not formally assessed (<10 studies per outcome); rationale reported in Main manuscript: Methods - Outcomes and Statistical Analysis. |
| Certainty of evidence | 22 | Present assessments of certainty (or confidence) in the body of evidence for each outcome assessed. | Main manuscript: Results - Risk of Bias and Certainty of Evidence; Supplementary Table S4. |
| **DISCUSSION** | | |  |
| Discussion | 23a | Provide a general interpretation of the results in the context of other evidence. | Main manuscript: Discussion (general interpretation of the findings in the context of other evidence). |
|  | 23b | Discuss any limitations of the evidence included in the review. | Main manuscript: Discussion (limitations of the included evidence). |
|  | 23c | Discuss any limitations of the review processes used. | Main manuscript: Discussion (limitations of the review process and analyses). |
|  | 23d | Discuss implications of the results for practice, policy, and future research. | Main manuscript: Discussion; Conclusion (implications and need for prospective comparative studies). |
| **OTHER INFORMATION** | | |  |
| Registration and protocol | 24a | Provide registration information for the review, including register name and registration number, or state that the review was not registered. | Main manuscript: Methods - Study Registration and Reporting (PROSPERO CRD420251286742). |
|  | 24b | Indicate where the review protocol can be accessed, or state that a protocol was not prepared. | Main manuscript: Methods - Study Registration and Reporting (a separate full protocol was not prepared). |
|  | 24c | Describe and explain any amendments to information provided at registration or in the protocol. | Main manuscript: Methods - Study Registration and Reporting; PROSPERO version history (amendments documented in the registration record). |
| Support | 25 | Describe sources of financial or non-financial support for the review, and the role of the funders or sponsors in the review. | Main manuscript: Statements & Declarations - Funding. |
| Competing interests | 26 | Declare any competing interests of review authors. | Main manuscript: Statements & Declarations - Conflict of Interest. |
| Availability of data, code and other materials | 27 | Report which of the following are publicly available and where they can be found: template data collection forms; data extracted from included studies; data used for all analyses; analytic code; any other materials used in the review. | Main manuscript: Statements & Declarations - Data Availability Statement (aggregated data available on reasonable request; code/forms not publicly deposited). |

*From:*  Page MJ, McKenzie JE, Bossuyt PM, Boutron I, Hoffmann TC, Mulrow CD, et al. The PRISMA 2020 statement: an updated guideline for reporting systematic reviews. BMJ 2021;372:n71. doi: 10.1136/bmj.n71. This work is licensed under CC BY 4.0. To view a copy of this license, visit <https://creativecommons.org/licenses/by/4.0/>
