# Supplementary material for: TACE-HAIC plus targeted therapy and immune checkpoint inhibitors for unresectable hepatocellular carcinoma: a systematic review and meta-analysis of TACE- and HAIC-based comparisons
Source: Front Oncol. 2026 Oct 23;16:1942009. doi: 10.3389/fonc.2026.1942009 (PMC13739493; doi:10.3389/fonc.2026.1942009)
Supplement: Supplementary file 2 [file Table1.docx]

**Supplementary Materials**

**Supplementary Table S1. Search strategies**

The searches covered database inception through August 19, 2026. Targeted therapy and immune checkpoint inhibitors were assessed during eligibility screening rather than required as mandatory search concepts. Subject headings and free-text terms were used where applicable.

Abbreviations: HCC, hepatocellular carcinoma; TACE, transarterial chemoembolization; HAIC, hepatic arterial infusion chemotherapy; TKI, tyrosine kinase inhibitor; ICI, immune checkpoint inhibitor; CNKI, China National Knowledge Infrastructure.

**PubMed**

| **#** | **Concept** | **Search terms** | **Results** |
| --- | --- | --- | --- |
| #1 | HCC | "Carcinoma, Hepatocellular"[Mesh] OR "Liver Neoplasms"[Mesh] OR "hepatocellular carcinoma"[Title/Abstract] OR "hepatocellular carcinomas"[Title/Abstract] OR HCC[Title/Abstract] OR hepatoma*[Title/Abstract] OR "liver cancer"[Title/Abstract] OR "liver carcinoma"[Title/Abstract] OR "primary liver cancer"[Title/Abstract] |  |
| #2 | TACE | "Chemoembolization, Therapeutic"[Mesh] OR "transarterial chemoembolization"[Title/Abstract] OR "transcatheter arterial chemoembolization"[Title/Abstract] OR "transarterial chemoembolisation"[Title/Abstract] OR "transcatheter arterial chemoembolisation"[Title/Abstract] OR TACE[Title/Abstract] OR cTACE[Title/Abstract] OR "c-TACE"[Title/Abstract] OR "DEB-TACE"[Title/Abstract] OR "drug-eluting bead TACE"[Title/Abstract] OR "conventional TACE"[Title/Abstract] |  |
| #3 | HAIC | "Infusions, Intra-Arterial"[Mesh] OR "hepatic arterial infusion chemotherapy"[Title/Abstract] OR "hepatic artery infusion chemotherapy"[Title/Abstract] OR "hepatic arterial infusion"[Title/Abstract] OR "hepatic artery infusion"[Title/Abstract] OR HAIC[Title/Abstract] OR "FOLFOX-HAIC"[Title/Abstract] OR "FOLFOX HAIC"[Title/Abstract] |  |
| #4 | Direct terminology | "TACE-HAIC"[Title/Abstract] OR "TACE HAIC"[Title/Abstract] |  |
| #5 | Final | #1 AND (#4 OR (#2 AND #3)) | 811 |

**Embase**

| **#** | **Concept** | **Search terms** | **Results** |
| --- | --- | --- | --- |
| #1 | HCC | 'liver cell carcinoma'/exp OR 'hepatocellular carcinoma':ti,ab,kw OR 'hepatocellular carcinomas':ti,ab,kw OR hcc:ti,ab,kw OR hepatoma*:ti,ab,kw OR 'liver cancer':ti,ab,kw OR 'liver carcinoma':ti,ab,kw OR 'primary liver cancer':ti,ab,kw |  |
| #2 | TACE | 'arterial chemoembolization'/exp OR 'chemoembolization'/exp OR 'transarterial chemoembolization':ti,ab,kw OR 'transcatheter arterial chemoembolization':ti,ab,kw OR 'transarterial chemoembolisation':ti,ab,kw OR 'transcatheter arterial chemoembolisation':ti,ab,kw OR tace:ti,ab,kw OR ctace:ti,ab,kw OR 'c-tace':ti,ab,kw OR 'deb-tace':ti,ab,kw OR 'drug-eluting bead tace':ti,ab,kw OR 'conventional tace':ti,ab,kw |  |
| #3 | HAIC | 'hepatic arterial infusion chemotherapy'/exp OR 'intraarterial drug administration'/exp OR 'hepatic arterial infusion chemotherapy':ti,ab,kw OR 'hepatic artery infusion chemotherapy':ti,ab,kw OR 'hepatic arterial infusion':ti,ab,kw OR 'hepatic artery infusion':ti,ab,kw OR haic:ti,ab,kw OR 'folfox-haic':ti,ab,kw OR 'folfox haic':ti,ab,kw |  |
| #4 | Direct terminology | 'tace-haic':ti,ab,kw OR 'tace haic':ti,ab,kw |  |
| #5 | Final | #1 AND (#4 OR (#2 AND #3)) | 1365 |

**Web of Science**

| **#** | **Concept** | **Search terms** | **Results** |
| --- | --- | --- | --- |
| #1 | HCC | TS=("hepatocellular carcinoma" OR "hepatocellular carcinomas" OR HCC OR hepatoma* OR "liver cancer" OR "liver carcinoma" OR "primary liver cancer") |  |
| #2 | TACE | TS=("transarterial chemoembolization" OR "transcatheter arterial chemoembolization" OR "transarterial chemoembolisation" OR "transcatheter arterial chemoembolisation" OR TACE OR cTACE OR "c-TACE" OR "DEB-TACE" OR "drug-eluting bead TACE" OR "conventional TACE") |  |
| #3 | HAIC | TS=("hepatic arterial infusion chemotherapy" OR "hepatic artery infusion chemotherapy" OR "hepatic arterial infusion" OR "hepatic artery infusion" OR HAIC OR "FOLFOX-HAIC" OR "FOLFOX HAIC") |  |
| #4 | Direct terminology | TS=("TACE-HAIC" OR "TACE HAIC") |  |
| #5 | Combined TACE/HAIC | #4 OR (#2 AND #3) |  |
| #6 | Final | #1 AND #5 | 641 |

**Cochrane Library**

| **#** | **Concept** | **Search terms** | **Results** |
| --- | --- | --- | --- |
| #1 | HCC subject terms | MeSH descriptor: [Carcinoma, Hepatocellular] explode all trees |  |
| #2 | HCC subject terms | MeSH descriptor: [Liver Neoplasms] explode all trees |  |
| #3 | HCC free-text | ("hepatocellular carcinoma" OR "hepatocellular carcinomas" OR HCC OR hepatoma* OR "liver cancer" OR "liver carcinoma" OR "primary liver cancer"):ti,ab,kw |  |
| #4 | TACE subject terms | MeSH descriptor: [Chemoembolization, Therapeutic] explode all trees |  |
| #5 | TACE free-text | ("transarterial chemoembolization" OR "transcatheter arterial chemoembolization" OR "transarterial chemoembolisation" OR "transcatheter arterial chemoembolisation" OR TACE OR cTACE OR "c-TACE" OR "DEB-TACE" OR "drug-eluting bead TACE" OR "conventional TACE"):ti,ab,kw |  |
| #6 | HAIC subject terms | MeSH descriptor: [Infusions, Intra-Arterial] explode all trees |  |
| #7 | HAIC free-text | ("hepatic arterial infusion chemotherapy" OR "hepatic artery infusion chemotherapy" OR "hepatic arterial infusion" OR "hepatic artery infusion" OR HAIC OR "FOLFOX-HAIC" OR "FOLFOX HAIC"):ti,ab,kw |  |
| #8 | Direct terminology | ("TACE-HAIC" OR "TACE HAIC"):ti,ab,kw |  |
| #9 | TACE AND HAIC | (#4 OR #5) AND (#6 OR #7) |  |
| #10 | Combined TACE/HAIC | #8 OR #9 |  |
| #11 | Final | (#1 OR #2 OR #3) AND #10 | 126 |

**CNKI**

| **#** | **Concept** | **Search terms** | **Results** |
| --- | --- | --- | --- |
| #1 | HCC | 篇关摘:(肝细胞癌 OR 肝癌 OR 原发性肝癌 OR HCC OR "hepatocellular carcinoma") |  |
| #2 | TACE | 篇关摘:(经导管动脉化疗栓塞 OR 经动脉化疗栓塞 OR 肝动脉化疗栓塞 OR TACE OR "transarterial chemoembolization") |  |
| #3 | HAIC | 篇关摘:(肝动脉灌注化疗 OR 肝动脉灌注 OR HAIC OR "hepatic arterial infusion chemotherapy") |  |
| #4 | Final | #1 AND #2 AND #3 | 478 |

**VIP**

| **#** | **Concept** | **Search terms** | **Results** |
| --- | --- | --- | --- |
| #1 | HCC | 肝细胞癌 OR 肝癌 OR 原发性肝癌 OR HCC OR "hepatocellular carcinoma" |  |
| #2 | TACE | 经导管动脉化疗栓塞 OR 经动脉化疗栓塞 OR 肝动脉化疗栓塞 OR TACE OR "transarterial chemoembolization" |  |
| #3 | HAIC | 肝动脉灌注化疗 OR 肝动脉灌注 OR HAIC OR "hepatic arterial infusion chemotherapy" |  |
| #4 | Final | #1 AND #2 AND #3 | 52 |

**WanFang Data**

| **#** | **Concept** | **Search terms** | **Results** |
| --- | --- | --- | --- |
| #1 | HCC | 肝细胞癌 OR 肝癌 OR 原发性肝癌 OR HCC OR "hepatocellular carcinoma" |  |
| #2 | TACE | 经导管动脉化疗栓塞 OR 经动脉化疗栓塞 OR 肝动脉化疗栓塞 OR TACE OR "transarterial chemoembolization" |  |
| #3 | HAIC | 肝动脉灌注化疗 OR 肝动脉灌注 OR HAIC OR "hepatic arterial infusion chemotherapy" |  |
| #4 | Final | #1 AND #2 AND #3 | 81 |

**SinoMed**

| **#** | **Concept** | **Search terms** | **Results** |
| --- | --- | --- | --- |
| #1 | HCC | 肝细胞癌 OR 肝癌 OR 原发性肝癌 OR HCC OR "hepatocellular carcinoma" (常用字段：智能) |  |
| #2 | TACE | 经导管动脉化疗栓塞 OR 经动脉化疗栓塞 OR 肝动脉化疗栓塞 OR TACE OR "transarterial chemoembolization" (常用字段：智能) |  |
| #3 | HAIC | 肝动脉灌注化疗 OR 肝动脉灌注 OR HAIC OR "hepatic arterial infusion chemotherapy" (常用字段：智能) |  |
| #4 | Final | #1 AND #2 AND #3 | 58 |

**Supplementary Table S2. NOS quality evaluation**

| **Study** | **Selection** | **Selection** | **Selection** | **Selection** | **Comparability** | **Outcome** | **Outcome** | **Outcome** | **Quality scores** |
| --- | --- | --- | --- | --- | --- | --- | --- | --- | --- |
|  | **Representativeness of the exposed cohort** | **Selection of the nonexposed cohort** | **Ascertainment of exposure** | **Demonstration that outcome of interest was not present at start of study** | **Comparability of cohorts on the basis of design or analysis** | **Assessment of outcome** | **Was follow-up long enough for outcomes to occur** | **Adequacy of follow-up of cohorts** |  |
| Chi 2025 | 1 | 1 | 1 | 1 | 2 | 1 | 1 | 0 | 8 |
| Huang 2024 | 1 | 1 | 1 | 1 | 1 | 1 | 1 | 0 | 7 |
| Feng 2026 | 1 | 1 | 1 | 1 | 2 | 1 | 1 | 0 | 8 |
| Li 2025 | 1 | 1 | 1 | 1 | 2 | 1 | 1 | 0 | 8 |
| Liu 2025 | 1 | 1 | 1 | 1 | 2 | 1 | 1 | 0 | 8 |
| Ni 2026 (JHC) | 1 | 1 | 1 | 1 | 1 | 1 | 1 | 0 | 7 |
| Wan 2025 | 1 | 1 | 1 | 1 | 2 | 1 | 1 | 0 | 8 |
| Wu 2025 | 1 | 1 | 1 | 1 | 2 | 1 | 1 | 0 | 8 |
| Zhou 2026 | 1 | 1 | 1 | 1 | 2 | 1 | 1 | 0 | 8 |
| Ma 2026 | 1 | 1 | 1 | 1 | 2 | 1 | 1 | 0 | 8 |
| Yang 2026 | 1 | 1 | 1 | 1 | 2 | 1 | 1 | 0 | 8 |
| Hu 2026 | 1 | 1 | 1 | 1 | 0 | 1 | 1 | 0 | 6 |
| Hou 2026 | 1 | 1 | 1 | 1 | 0 | 1 | 1 | 0 | 6 |
| Ni 2026 (BMC Cancer) | 1 | 1 | 1 | 1 | 1 | 1 | 1 | 0 | 7 |

Note: NOS, Newcastle-Ottawa Scale. Selection contains four items (maximum 4 points), Comparability contains one item (maximum 2 points), and Outcome contains three items (maximum 3 points); the maximum score is 9. NOS was retained as a supplementary descriptive assessment, whereas ROBINS-I was used to assess risk of bias for the survival outcomes. All included studies were assessed using the same NOS scoring convention.

**Supplementary Table S3. ROBINS-I (2016) assessment for survival outcomes**

D1, bias due to confounding; D2, bias in selection of participants; D3, bias in classification of interventions; D4, bias due to deviations from intended interventions; D5, bias due to missing data; D6, bias in measurement of outcomes; D7, bias in selection of the reported result.

| **Study** | **Comparison** | **D1** | **D2** | **D3** | **D4** | **D5** | **D6** | **D7** | **Overall** |
| --- | --- | --- | --- | --- | --- | --- | --- | --- | --- |
| **Panel A. Overall survival** | | | | | | | | | |
| Chi 2025 | Both | Serious | Moderate | Low | Low | Low | Low | Moderate | Serious |
| Huang 2024 | TACE | Moderate | Moderate | Low | Low | Low | Low | Moderate | Moderate |
| Feng 2026 | TACE | Moderate | Moderate | Low | Low | Low | Low | Moderate | Moderate |
| Li 2025 | TACE | Moderate | Serious | Moderate | Moderate | Low | Low | Moderate | Serious |
| Liu 2025 | HAIC | Moderate | Moderate | Low | Low | Low | Low | Moderate | Moderate |
| Ni 2026 (JHC) | TACE | Serious | Moderate | Low | Low | Low | Low | Moderate | Serious |
| Wan 2025 | TACE | Moderate | Moderate | Low | Low | Moderate | Low | Low | Moderate |
| Wu 2025 | HAIC | Moderate | Moderate | Low | Low | Low | Low | Moderate | Moderate |
| Zhou 2026 | HAIC | Moderate | Moderate | Low | Low | Low | Low | Moderate | Moderate |
| Ma 2026 | TACE | Moderate | Low | Low | Low | Low | Low | Moderate | Moderate |
| Yang 2026 | TACE | Moderate | Serious | Low | Low | Low | Low | Moderate | Serious |
| Hu 2026 | TACE | Serious | Serious | Low | Low | Low | Low | Moderate | Serious |
| Hou 2026 | TACE | Serious | Moderate | Low | Low | Moderate | Low | Moderate | Serious |
| Ni 2026 (BMC Cancer) | HAIC | Serious | Moderate | Low | Low | Low | Low | Moderate | Serious |
| **Panel B. Progression-free survival** | | | | | | | | | |
| Chi 2025 | Both | Serious | Moderate | Low | Low | Low | Moderate | Moderate | Serious |
| Feng 2026 | TACE | Moderate | Moderate | Low | Low | Low | Moderate | Moderate | Moderate |
| Li 2025 | TACE | Moderate | Serious | Moderate | Moderate | Low | Moderate | Moderate | Serious |
| Liu 2025 | HAIC | Moderate | Moderate | Low | Low | Low | Moderate | Moderate | Moderate |
| Ni 2026 (JHC) | TACE | Serious | Moderate | Low | Low | Low | Moderate | Low | Serious |
| Wan 2025 | TACE | Moderate | Moderate | Low | Low | Moderate | Moderate | Low | Moderate |
| Wu 2025 | HAIC | Moderate | Moderate | Low | Low | Low | Moderate | Moderate | Moderate |
| Ma 2026 | TACE | Moderate | Low | Low | Low | Low | Moderate | Moderate | Moderate |
| Yang 2026 | TACE | Moderate | Serious | Low | Low | Low | Moderate | Moderate | Serious |
| Hu 2026 | TACE | Serious | Serious | Low | Low | Low | Moderate | Moderate | Serious |
| Hou 2026 | TACE | Serious | Moderate | Low | Low | Moderate | Moderate | Moderate | Serious |
| Ni 2026 (BMC Cancer) | HAIC | Serious | Moderate | Low | Low | Low | Moderate | Moderate | Serious |

The target-trial time zero was initiation of the index treatment strategy. For Chi 2025, the same domain judgments were applied to its TACE-based and HAIC-based direct comparisons. Huang 2024 and Zhou 2026 were not included in the PFS panel because no eligible PFS HR was synthesized. Hu 2026 reported survival outcomes but did not contribute a pooled survival HR. ROBINS-I judgments here refer primarily to survival outcomes.

Main Serious-risk concerns were: unadjusted direct comparisons and baseline imbalance (Chi 2025); participant selection requiring complete longitudinal AFP/follow-up data and heterogeneous treatment timing despite IPTW (Li 2025); residual confounding in the unmatched TACE comparison and use of unadjusted direct survival estimates (Ni 2026 [JHC]); time-zero misalignment because OS/PFS began at HCC diagnosis (Yang 2026); unmatched complete-data selection and residual confounding (Hu 2026); an unmatched TACE-HAIC-TP versus TACE-TP comparison without dedicated propensity-score adjustment (Hou 2026); and an unmatched HAIC-based comparison with residual confounding despite prognostic modeling (Ni 2026 [BMC Cancer]).

**Supplementary Table S4. GRADE summary of findings and certainty of evidence**

| **Outcome** | **Studies (participants)** | **Relative effect (95% CI)** | **Absolute difference per 1000** | **I² (%)** | **Certainty (GRADE)** |
| --- | --- | --- | --- | --- | --- |
| **Panel A. TACE-based comparison** | | | | | |
| Overall survival | 9 (1,510) | HR 0.59 (0.47-0.75) | Not estimable | 54.7 | Low (a,f) |
| Progression-free survival | 8 (1,387) | HR 0.65 (0.55-0.78) | Not estimable | 36.2 | Low (a,f) |
| Objective response rate | 9 (1,443) | RR 1.34 (1.19-1.52) | 134 more (75 to 205 more) | 0.3 | Very low (a,b) |
| Disease control rate | 9 (1,443) | RR 1.14 (1.07-1.21) | 114 more (57 to 170 more) | 50.5 | Very low (a,b) |
| Complete response | 9 (1,443) | RR 2.17 (1.46-3.21) | 52 more (20 to 98 more) | 0.0 | Very low (a,b,e) |
| Overall grade ≥3 TRAEs | 4 (455)* | RR 1.16 (0.81-1.67) | 29 more (35 fewer to 123 more) | 0.0 | Very low (a,b,d) |
| **Panel B. HAIC-based comparison** | | | | | |
| Overall survival | 5 (572) | HR 0.56 (0.43-0.72) | Not estimable | 0.0 | Low (a,f) |
| Progression-free survival | 4 (479) | HR 0.64 (0.48-0.86) | Not estimable | 42.3 | Very low (a,b,c,d) |
| Objective response rate | 5 (572) | RR 1.24 (1.03-1.50) | 123 more (15 to 255 more) | 27.5 | Very low (a,d) |
| Disease control rate | 5 (572) | RR 1.09 (0.99-1.19) | 72 more (8 fewer to 153 more) | 33.4 | Very low (a,d) |
| Complete response | 5 (572) | RR 1.62 (0.61-4.30) | 15 more (9 fewer to 80 more) | 0.0 | Very low (a,e) |
| Overall grade ≥3 TRAEs | 3 (249) | RR 1.27 (0.92-1.74) | 89 more (26 fewer to 245 more) | 0.0 | Very low (a,d) |

* Four studies reported overall grade ≥3 TRAEs in the TACE-based comparison; one was a double-zero study, leaving three informative studies for the relative-effect estimate.

(a) Observational evidence started at low certainty under the conventional GRADE framework.

(b) Downgraded for serious risk of bias because residual confounding or selection remained plausible.

(c) Downgraded for inconsistency because moderate heterogeneity and variation in study-specific estimates remained.

(d) Downgraded for imprecision because the confidence interval included no effect and clinically important benefit and/or harm, or because the evidence was based on very few informative studies.

(e) Certainty was very low because complete-response events were sparse and the confidence interval was very wide.

(f) No additional downgrade for risk of bias was applied because sensitivity analyses excluding studies at serious overall ROBINS-I risk yielded pooled estimates that remained consistent in direction with the main analyses.

HR, hazard ratio; RR, risk ratio; CI, confidence interval; TRAEs, treatment-related adverse events. RR>1 for efficacy outcomes favors TACE-HAIC; RR>1 for TRAEs indicates greater toxicity with TACE-HAIC.

**Supplementary Table S5. Follow-up and source of survival estimates used in the meta-analysis.**

| **Study** | **Comparison** | **Analysis population** | **Reported follow-up** | **OS HR source** | **PFS HR source** | **Adjustment / extraction note** |
| --- | --- | --- | --- | --- | --- | --- |
| Chi 2025 | Both | Original/analysis: LPTH 24; LPT 38; LPH 12 | 16.1 mo (IQR 1.6-43.8) | KM reconstructed | KM reconstructed | Digitized Kaplan-Meier curves plus numbers at risk (Guyot method); unadjusted direct comparisons. |
| Huang 2024 | TACE | Original/analysis: 63 vs 60 | 11 mo (range 2-36) | Reported Cox HR | Not synthesized | Multivariable Cox-adjusted OS estimate; cohort itself was unmatched. |
| Feng 2026 | TACE | Original 156 vs 118; efficacy/survival PSM 107 vs 107; safety original 156 vs 118 | 34.0 vs 33.8 mo | Reported post-PSM HR | Reported post-PSM HR | 1:1 PSM for efficacy/survival; safety used original cohort. |
| Li 2025 | TACE | Original 154 vs 305; stabilized IPTW for survival; raw counts for response/safety | 29.9 mo (95% CI 27.5-32.3) | Directly reported stabilized-IPTW HR (Suppl Fig S1) | Directly reported stabilized-IPTW HR (Suppl Fig S1) | Stabilized-IPTW treatment HRs were used for survival. Response/safety RRs were calculated from raw event counts. IPTW pseudo-populations were not counted as additional patients. |
| Liu 2025 | HAIC | Original 103 vs 94; post-PSM 50 vs 50 | 13.1 vs 14.3 mo | Reported post-PSM HR | Reported post-PSM HR | 1:1 PSM; post-PSM efficacy/survival/safety data used. Published post-PSM extrahepatic-metastasis counts were internally inconsistent with the pre-PSM cohort and were not reproduced in Table 1a. |
| Ni 2026 (JHC) | TACE | Original/analysis: 103 vs 118 | Mean 23.8+/-11.2 vs 18.4+/-8.7 mo | Reported KM comparison HR | Reported KM comparison HR | Unmatched cohort; direct treatment-comparison KM HRs were unadjusted. A separate multivariable prognostic model included post-treatment conversion resection and was not used as the treatment-effect estimate. |
| Wan 2025 | TACE | Original 102 vs 56; efficacy/survival PSM 35 vs 35; safety original 102 vs 56 | 16.25 mo overall | Post-PSM HR from Supplementary Fig. 5 | Post-PSM HR from Supplementary Fig. 6 | 1:1 PSM for efficacy/survival; safety used original cohort. Source direction (triple vs quadruple) inverted to the common meta-analysis direction. |
| Wu 2025 | HAIC | Original 119 vs 244; post-PSM 87 vs 143 | 17.30 vs 24.07 mo (P<0.001) | Reported post-PSM HR | Reported post-PSM HR | Post-PSM estimates used. ECOG 0-2 was an eligibility criterion but post-PSM ECOG category counts were not reported. The published OS HR and 95% CI were extracted as reported. The standard error used for inverse-variance pooling was derived from the reported 95% CI on the log scale; therefore, the symmetric model-based confidence limits displayed in the forest plot may differ from the originally reported limits. |
| Zhou 2026 | HAIC | Original 41 vs 172; post-PSM 31 vs 62 | NR | Post-PSM multivariable Cox HR | Not synthesized | 1:2 PSM; post-PSM OS estimate and response/safety population used. |
| Ma 2026 | TACE | Original 58 vs 64; IPTW for survival; raw counts for response/safety | 18 vs 14 mo | Post-IPTW HR | Post-IPTW HR | IPTW survival estimates used; response/safety RRs were calculated from raw event counts. |
| Yang 2026 | TACE | Original 42 vs 72; post-PSM 35 vs 35 | 17 mo | Reported post-PSM HR | Reported post-PSM HR | 1:1 PSM; OS/PFS were measured from HCC diagnosis rather than treatment initiation. |
| Hu 2026 | TACE | Original/analysis: 48 vs 54; response/safety raw | 19.5 mo (range 6.2-35.8) | No usable treatment HR | No usable treatment HR | Median OS/PFS and log-rank P values reported; retained for qualitative survival synthesis only. Full cohort used for response and safety. |
| Hou 2026 | TACE | Original/analysis: 79 vs 90 | Follow-up schedule reported; median follow-up NR | Reported direct-comparison HR | Reported direct-comparison HR | Unmatched TACE-HAIC-TP versus TACE-TP comparison; direct treatment HRs used. The HAIC-based comparison from Hou 2026 was excluded because its patient population overlapped with that of Liu 2025; Liu 2025 was retained to avoid double counting within the HAIC-based synthesis. |
| Ni 2026 (BMC Cancer) | HAIC | Original/analysis: 51 vs 62 | 17.6 mo (range 6.5–41.0) vs 16.0 mo (range 6.5–30.5); follow-up every 4–6 weeks; cutoff December 31, 2025 | Reported direct KM HR | Reported direct KM HR | Multicenter unmatched comparison. Direct Kaplan-Meier treatment HRs were used; multivariable prognostic-model estimates were not substituted for the direct treatment comparison. |

HR, hazard ratio; PSM, propensity score matching; IPTW, inverse probability of treatment weighting; KM, Kaplan-Meier; NR, not reported. 'vs' follows the intervention-versus-control order used in the corresponding study where group-specific follow-up was reported. Original cohort sizes refer to actual enrolled patients; weighted pseudo-populations generated by IPTW are not patient counts. Outcome-specific analytic populations are shown explicitly because matched/weighted survival analyses and raw binary response/safety analyses sometimes used different denominators.

**Supplementary Table S6a. Pooled analyses of individual adverse events in the TACE-based comparison.**

| **AE** | **Grade** | **Informative studies** | **TACE-HAIC events/participants** | **Comparator events/participants** | **RR (95% CI)** | **P** | **I² (%)** |
| --- | --- | --- | --- | --- | --- | --- | --- |
| ALT elevation | Any grade | 6 | 288/557 | 401/641 | 1.00 (0.85-1.19) | 1.000 | 59.9 |
|  | Grade ≥3 | 5 | 55/494 | 93/581 | 0.92 (0.68-1.25) | 0.591 | 0.0 |
| AST elevation | Any grade | 6 | 328/557 | 461/641 | 0.93 (0.84-1.03) | 0.163 | 29.8 |
|  | Grade ≥3 | 5 | 91/494 | 156/581 | 0.96 (0.74-1.25) | 0.760 | 0.0 |
| Hypoalbuminemia | Any grade | 4 | 94/264 | 60/247 | 1.49 (1.14-1.96) | 0.004 | 9.3 |
| Leukopenia | Any grade | 7 | 199/592 | 208/676 | 1.11 (0.94-1.31) | 0.218 | 0.0 |
|  | Grade ≥3 | 4 | 24/470 | 25/543 | 1.15 (0.65-2.03) | 0.630 | 4.8 |
| Hyperbilirubinemia | Any grade | 8 | 212/695 | 243/794 | 1.11 (0.95-1.28) | 0.170 | 0.0 |
|  | Grade ≥3 | 6 | 28/543 | 33/697 | 1.11 (0.67-1.83) | 0.684 | 0.0 |
| Proteinuria | Any grade | 4 | 78/392 | 107/525 | 1.14 (0.88-1.46) | 0.310 | 0.0 |
|  | Grade ≥3 | 5 | 5/440 | 11/579 | 1.14 (0.32-4.00) | 0.839 | 0.0 |
| Nausea | Any grade | 7 | 286/660 | 225/759 | 1.50 (1.17-1.93) | 0.001 | 71.7 |
| Vomiting | Any grade | 6 | 188/558 | 147/703 | 1.55 (1.31-1.84) | <0.001 | 0.0 |
| Decreased appetite | Any grade | 3 | 140/293 | 89/272 | 1.54 (1.17-2.03) | 0.002 | 45.1 |
| Fever | Any grade | 7 | 214/660 | 279/759 | 0.88 (0.59-1.29) | 0.522 | 80.1 |
|  | Grade ≥3 | 3 | 11/334 | 8/461 | 2.35 (0.33-16.60) | 0.393 | 67.4 |
| Fatigue | Any grade | 8 | 212/739 | 242/849 | 0.93 (0.72-1.20) | 0.578 | 51.8 |
| Abdominal pain | Any grade | 9 | 424/774 | 472/884 | 1.08 (0.90-1.30) | 0.412 | 65.5 |
|  | Grade ≥3 | 4 | 33/515 | 35/597 | 0.80 (0.51-1.26) | 0.333 | 0.0 |
| Diarrhea | Any grade | 7 | 111/557 | 113/519 | 1.02 (0.82-1.28) | 0.862 | 12.6 |
|  | Grade ≥3 | 3 | 15/283 | 14/274 | 1.07 (0.53-2.16) | 0.850 | 0.0 |
| Hypertension | Any grade | 6 | 92/558 | 113/703 | 0.88 (0.65-1.18) | 0.401 | 30.5 |
|  | Grade ≥3 | 6 | 29/543 | 24/697 | 1.28 (0.75-2.17) | 0.362 | 0.0 |
| Hypothyroidism | Any grade | 6 | 62/558 | 79/703 | 1.06 (0.78-1.45) | 0.713 | 0.0 |
|  | Grade ≥3 | 3 | 4/238 | 4/220 | 1.07 (0.27-4.13) | 0.923 | 0.0 |
| Rash | Any grade | 7 | 94/636 | 101/731 | 1.01 (0.79-1.30) | 0.938 | 0.0 |
| Anemia | Any grade | 4 | 149/315 | 248/434 | 0.98 (0.79-1.21) | 0.853 | 18.8 |
|  | Grade ≥3 | 3 | 18/280 | 37/399 | 0.87 (0.42-1.81) | 0.709 | 25.5 |
| Hand-foot syndrome | Any grade | 8 | 145/711 | 151/824 | 0.97 (0.80-1.17) | 0.753 | 10.0 |
|  | Grade ≥3 | 7 | 26/645 | 29/753 | 0.92 (0.55-1.56) | 0.754 | 0.0 |
| Weight loss | Any grade | 4 | 91/317 | 84/310 | 1.06 (0.83-1.36) | 0.644 | 0.0 |
| Gastrointestinal bleeding | Any grade | 4 | 26/471 | 32/605 | 1.04 (0.62-1.74) | 0.882 | 0.0 |
|  | Grade ≥3 | 3 | 8/413 | 15/541 | 0.78 (0.33-1.85) | 0.572 | 0.0 |
| Thrombocytopenia | Any grade | 7 | 225/592 | 302/676 | 0.92 (0.79-1.07) | 0.281 | 20.5 |
|  | Grade ≥3 | 6 | 44/542 | 78/635 | 0.81 (0.54-1.24) | 0.320 | 0.0 |
| Neutropenia | Grade ≥3 | 3 | 15/358 | 22/477 | 1.12 (0.59-2.15) | 0.731 | 0.0 |

P values refer to the pooled treatment effect; heterogeneity was quantified using I². Only grade levels with at least three informative studies are shown; levels with fewer than three informative studies are omitted rather than displayed as empty pooled rows. Events/participants are summed across informative studies only; double-zero studies do not contribute to pooled estimates. Study-level data for outcomes not quantitatively synthesized are shown in Supplementary Table S7. Individual-AE analyses are exploratory.

**Supplementary Table S6b. Pooled analyses of individual adverse events in the HAIC-based comparison.**

| **AE** | **Grade** | **Informative studies** | **TACE-HAIC events/participants** | **Comparator events/participants** | **RR (95% CI)** | **P** | **I² (%)** |
| --- | --- | --- | --- | --- | --- | --- | --- |
| ALT elevation | Any grade | 3 | 101/142 | 93/216 | 1.47 (1.14-1.90) | 0.003 | 42.0 |
|  | Grade ≥3 | 3 | 36/142 | 21/216 | 2.32 (1.41-3.81) | <0.001 | 0.4 |
| AST elevation | Any grade | 4 | 122/192 | 120/266 | 1.31 (0.99-1.73) | 0.058 | 57.2 |
|  | Grade ≥3 | 4 | 40/192 | 30/266 | 1.82 (1.19-2.80) | 0.006 | 10.0 |
| Hypoalbuminemia | Any grade | 5 | 91/243 | 98/328 | 1.31 (1.04-1.64) | 0.020 | 0.0 |
| Hyperbilirubinemia | Any grade | 5 | 77/243 | 81/328 | 1.34 (0.99-1.80) | 0.055 | 0.0 |
|  | Grade ≥3 | 4 | 14/212 | 9/267 | 1.95 (0.86-4.42) | 0.110 | 0.0 |
| Proteinuria | Any grade | 3 | 11/125 | 13/124 | 0.80 (0.36-1.74) | 0.579 | 0.0 |
| Vomiting | Any grade | 3 | 71/162 | 95/217 | 1.03 (0.77-1.38) | 0.843 | 13.5 |
| Fever | Any grade | 3 | 72/162 | 61/217 | 1.45 (1.02-2.07) | 0.040 | 28.5 |
| Fatigue | Any grade | 3 | 75/162 | 86/217 | 1.09 (0.91-1.30) | 0.344 | 0.0 |
| Abdominal pain | Any grade | 4 | 123/212 | 91/267 | 1.61 (1.09-2.39) | 0.017 | 67.4 |
|  | Grade ≥3 | 3 | 19/188 | 7/255 | 3.06 (1.32-7.12) | 0.009 | 0.0 |
| Diarrhea | Any grade | 4 | 40/212 | 35/267 | 1.30 (0.86-1.96) | 0.212 | 0.0 |
|  | Grade ≥3 | 3 | 9/188 | 6/255 | 1.85 (0.66-5.15) | 0.240 | 0.0 |
| Hypertension | Any grade | 4 | 84/212 | 108/267 | 1.05 (0.80-1.38) | 0.726 | 0.0 |
|  | Grade ≥3 | 4 | 22/212 | 24/267 | 1.15 (0.66-2.01) | 0.623 | 0.0 |
| Hypothyroidism | Any grade | 3 | 35/162 | 45/217 | 1.06 (0.71-1.57) | 0.773 | 0.0 |
| Rash | Any grade | 3 | 20/125 | 13/124 | 1.36 (0.72-2.60) | 0.348 | 0.0 |
| Hand-foot syndrome | Any grade | 3 | 31/125 | 29/124 | 1.04 (0.67-1.61) | 0.861 | 0.0 |
|  | Grade ≥3 | 3 | 6/125 | 5/124 | 1.17 (0.38-3.62) | 0.785 | 0.0 |
| Weight loss | Any grade | 3 | 40/161 | 59/205 | 0.96 (0.69-1.35) | 0.812 | 0.0 |
| Thrombocytopenia | Any grade | 3 | 74/161 | 109/205 | 0.90 (0.73-1.11) | 0.324 | 0.0 |
|  | Grade ≥3 | 3 | 25/161 | 29/205 | 1.28 (0.79-2.07) | 0.315 | 0.0 |

P values refer to the pooled treatment effect; heterogeneity was quantified using I². Only grade levels with at least three informative studies are shown; levels with fewer than three informative studies are omitted rather than displayed as empty pooled rows. Events/participants are summed across informative studies only; double-zero studies do not contribute to pooled estimates. Study-level data for outcomes not quantitatively synthesized are shown in Supplementary Table S7. Individual-AE analyses are exploratory.

**Supplementary Table S7. Study-level data for specific adverse events not quantitatively synthesized because fewer than three studies provided informative comparative data.**

| **AE** | **Study** | **TACE-HAIC events/n** | **Comparator events/n** |
| --- | --- | --- | --- |
| **TACE-based comparison** | | | |
| Hypoalbuminemia (Grade ≥3) | Ni 2026 (JHC) | 0/103 | 0/118 |
|  | Wan 2025 | 0/102 | 0/56 |
|  | Chi 2025 | 0/24 | 0/38 |
| Nausea (Grade ≥3) | Feng 2026 | 16/156 | 10/118 |
|  | Ni 2026 (JHC) | 0/103 | 0/118 |
|  | Wan 2025 | 0/102 | 0/56 |
|  | Ma 2026 | 0/58 | 0/64 |
|  | Chi 2025 | 0/24 | 0/38 |
| Vomiting (Grade ≥3) | Feng 2026 | 13/156 | 6/118 |
|  | Ni 2026 (JHC) | 0/103 | 0/118 |
|  | Chi 2025 | 0/24 | 0/38 |
| Fatigue (Grade ≥3) | Feng 2026 | 4/156 | 6/118 |
|  | Ni 2026 (JHC) | 0/103 | 0/118 |
|  | Wan 2025 | 0/102 | 0/56 |
|  | Ma 2026 | 0/58 | 0/64 |
|  | Chi 2025 | 0/24 | 0/38 |
|  | Hu 2026 | 3/48 | 2/54 |
| Rash (Grade ≥3) | Feng 2026 | 11/156 | 7/118 |
|  | Li 2025 | 2/154 | 3/305 |
|  | Wan 2025 | 0/102 | 0/56 |
|  | Ma 2026 | 0/58 | 0/64 |
|  | Chi 2025 | 0/24 | 0/38 |
| Weight loss (Grade ≥3) | Feng 2026 | 7/156 | 7/118 |
|  | Ma 2026 | 0/58 | 0/64 |
|  | Chi 2025 | 0/24 | 0/38 |
| Neutropenia (Any grade) | Feng 2026 | 11/156 | 5/118 |
|  | Li 2025 | 48/154 | 79/305 |
| **HAIC-based comparison** | | | |
| Hypoalbuminemia (Grade ≥3) | Liu 2025 | 3/50 | 1/50 |
|  | Wu 2025 | 8/87 | 8/143 |
|  | Zhou 2026 | 0/31 | 0/61 |
|  | Chi 2025 | 0/24 | 0/12 |
|  | Ni 2026 (BMC Cancer) | 0/51 | 0/62 |
| Leukopenia (Any grade) | Chi 2025 | 10/24 | 4/12 |
| Leukopenia (Grade ≥3) | Chi 2025 | 0/24 | 0/12 |
| Proteinuria (Grade ≥3) | Liu 2025 | 1/50 | 0/50 |
|  | Chi 2025 | 1/24 | 0/12 |
|  | Ni 2026 (BMC Cancer) | 0/51 | 0/62 |
| Nausea (Any grade) | Chi 2025 | 9/24 | 4/12 |
|  | Ni 2026 (BMC Cancer) | 46/51 | 52/62 |
| Nausea (Grade ≥3) | Chi 2025 | 0/24 | 0/12 |
|  | Ni 2026 (BMC Cancer) | 0/51 | 0/62 |
| Fever (Grade ≥3) | Wu 2025 | 5/87 | 4/143 |
|  | Chi 2025 | 4/24 | 0/12 |
|  | Ni 2026 (BMC Cancer) | 0/51 | 0/62 |
| Fatigue (Grade ≥3) | Wu 2025 | 2/87 | 5/143 |
|  | Chi 2025 | 0/24 | 0/12 |
|  | Ni 2026 (BMC Cancer) | 0/51 | 0/62 |
| Hypothyroidism (Grade ≥3) | Wu 2025 | 4/87 | 9/143 |
|  | Chi 2025 | 0/24 | 0/12 |
|  | Ni 2026 (BMC Cancer) | 1/51 | 3/62 |
| Vomiting (Grade ≥3) | Wu 2025 | 7/87 | 9/143 |
|  | Chi 2025 | 0/24 | 0/12 |
|  | Ni 2026 (BMC Cancer) | 0/51 | 0/62 |
| Rash (Grade ≥3) | Liu 2025 | 1/50 | 2/50 |
|  | Chi 2025 | 0/24 | 0/12 |
|  | Ni 2026 (BMC Cancer) | 0/51 | 0/62 |
| Anemia (Any grade) | Wu 2025 | 8/87 | 16/143 |
|  | Chi 2025 | 14/24 | 5/12 |
| Anemia (Grade ≥3) | Wu 2025 | 1/87 | 2/143 |
|  | Chi 2025 | 3/24 | 0/12 |
| Weight loss (Grade ≥3) | Liu 2025 | 0/50 | 0/50 |
|  | Wu 2025 | 5/87 | 4/143 |
|  | Chi 2025 | 0/24 | 0/12 |
| Neutropenia (Any grade) | Wu 2025 | 35/87 | 56/143 |
| Neutropenia (Grade ≥3) | Wu 2025 | 7/87 | 14/143 |
| Gastrointestinal bleeding (Any grade) | Ni 2026 (BMC Cancer) | 3/51 | 2/62 |
| Gastrointestinal bleeding (Grade ≥3) | Ni 2026 (BMC Cancer) | 3/51 | 2/62 |

An informative study had at least one event in either treatment group. Double-zero studies were not counted as informative. Outcomes with fewer than three informative studies were not quantitatively pooled; all reporting studies for those outcomes, including double-zero studies, are listed below to make the available event counts and denominators explicit.

Exploratory meta-regression and formal publication-bias testing were not performed because no comparator-specific outcome included at least 10 studies.

Supplementary Table S8. Sensitivity analyses of efficacy outcomes.

| **Comparison** | **Outcome** | **Sensitivity analysis** | **Studies** | **Pooled effect (95% CI)** | **I² (%)** |
| --- | --- | --- | --- | --- | --- |
| TACE-based | OS | Exclude studies at serious overall ROBINS-I risk | 4 | HR 0.45 (0.32-0.63) | 27.6 |
| TACE-based | PFS | Exclude studies at serious overall ROBINS-I risk | 3 | HR 0.53 (0.41-0.67) | 0.0 |
| TACE-based | OS | Exclude Li 2025 | 8 | HR 0.55 (0.45-0.69) | 36.7 |
| TACE-based | PFS | Exclude Li 2025 | 7 | HR 0.61 (0.52-0.71) | 0.7 |
| TACE-based | ORR | Exclude Li 2025 | 8 | RR 1.42 (1.26-1.60) | 0.0 |
| TACE-based | DCR | Exclude Li 2025 | 8 | RR 1.16 (1.10-1.22) | 0.0 |
| TACE-based | CR | Exclude Li 2025 | 8 reports | RR 1.88 (1.23-2.86) | 0.0 |
| TACE-based | OS | Leave-one-out range | 8 per analysis | HR 0.55-0.64; all statistically significant | 36.7-59.8 |
| TACE-based | PFS | Leave-one-out range | 7 per analysis | HR 0.61-0.69; all statistically significant | 0.7-45.1 |
| TACE-based | ORR | Leave-one-out range | 8 per analysis | RR 1.29-1.42; all statistically significant | 0.0-12.3 |
| TACE-based | DCR | Leave-one-out range | 8 per analysis | RR 1.12-1.16; all statistically significant | 0.0-56.1 |
| TACE-based | CR | Leave-one-out range | 8 per analysis | RR 1.88-2.40; all statistically significant | 0.0-8.5 |
| HAIC-based | OS | Exclude studies at serious overall ROBINS-I risk | 3 | HR 0.56 (0.40-0.78) | 1.5 |
| HAIC-based | PFS | Exclude studies at serious overall ROBINS-I risk | 2 | HR 0.61 (0.36-1.04) | 72.6 |
| HAIC-based | OS | Leave-one-out range | 4 per analysis | HR 0.52-0.59; all statistically significant | 0.0 |
| HAIC-based | PFS | Omit Wu 2025 | 3 | HR 0.56 (0.42-0.75) | 32.0 |
| HAIC-based | PFS | Omit Liu 2025 | 3 | HR 0.72 (0.56-0.92) | 6.7 |
| HAIC-based | PFS | Omit Chi 2025 | 3 | HR 0.61 (0.45-0.84) | 47.8 |
| HAIC-based | PFS | Omit Ni 2026 (BMC Cancer) | 3 | HR 0.68 (0.42-1.09) | 58.9 |
| HAIC-based | ORR | Omit Liu 2025 | 4 | RR 1.22 (0.96-1.55) | 43.4 |
| HAIC-based | ORR | Omit Wu 2025 | 4 | RR 1.33 (1.06-1.69) | 19.7 |
| HAIC-based | ORR | Omit Zhou 2026 | 4 | RR 1.30 (1.06-1.59) | 15.0 |
| HAIC-based | ORR | Omit Chi 2025 | 4 | RR 1.22 (0.99-1.51) | 40.9 |
| HAIC-based | ORR | Omit Ni 2026 (BMC Cancer) | 4 | RR 1.14 (0.96-1.35) | 0.0 |
| HAIC-based | DCR | Leave-one-out range | 4 per analysis | RR 1.04-1.12; all nonsignificant | 0.0-48.6 |
| HAIC-based | CR | Leave-one-out range | 4 per analysis | RR 1.31-2.06; all nonsignificant | 0.0-2.0 |

Note: Leave-one-out ranges summarize the minimum and maximum pooled estimates across single-study omissions. For HAIC-based PFS and ORR, individual omissions are shown because statistical significance was sensitive to specific studies. Serious-risk exclusions refer to the overall ROBINS-I judgment for the corresponding survival outcome.

Supplementary Table S9. Reports excluded after detailed eligibility assessment and primary reasons for exclusion

One primary exclusion reason was assigned per report. Where more than one reason applied, the first decisive eligibility criterion was recorded. Overlapping/superseded cohorts were excluded to avoid double counting within the relevant comparator-specific synthesis. Theses/dissertations and other non-peer-reviewed reports were excluded under the final amended eligibility criteria.

| **No.** | **Report** | **Year** | **Identifier / source** | **Primary reason for exclusion** |
| --- | --- | --- | --- | --- |
| 1 | Shindoh J. et al. Prognostic Benefit of Sequential Locoregional Interventions During First-Line Systemic Therapy for Hepatocellular Carcinoma: Insights from Interaction Analysis of Lenvatinib and Atezolizumab/Bevacizumab Annals of Surgical Oncology | 2026 | 10.1245/s10434-026-20226-x | Outcome/subgroup not separately extractable: Comparator-specific TACE-HAIC versus TACE/HAIC treatment effects were not separately extractable from the heterogeneous locoregional-intervention groups. |
| 2 | Gao F. et al. Survival Benefit of Combined Systemic and Locoregional Therapy in Hepatocellular Carcinoma with Portal Vein Tumor Thrombus: A Propensity Score-Matched Analysis Liver Cancer | 2026 | 10.1159/000551935 | Ineligible intervention: Compared TACE or HAIC plus systemic therapy with systemic therapy alone; no prespecified TACE-HAIC intervention arm. |
| 3 | Ou et al. Multimodal therapeutic efficacy model for predicting early treatment response to TACE-HAIC combined with immune checkpoint inhibitors and tyrosine kinase inhibitors in unresectable hepatocellular carcinoma EUROPEAN JOURNAL OF RADIOLOGY | 2026 | 10.1016/j.ejrad.2026.112784 | No eligible comparative arm: Prediction-model study in patients receiving TACE-HAIC plus targeted therapy and ICIs; no eligible treatment comparator. |
| 4 | Li et al. Retrospective analysis of transarterial chemoembolization or hepatic arterial infusion chemotherapy combined with lenvatinib with or without PD-1 inhibitor as first-line therapy for unresectable hepatocellular carcinoma with high tumor burden: a propensity score-matched study FRONTIERS IN IMMUNOLOGY | 2026 | 10.3389/fimmu.2026.1717797 | Ineligible intervention: Compared TACE- or HAIC-based regimens with lenvatinib with/without PD-1 inhibition; no TACE-HAIC intervention arm. |
| 5 | Chen et al. Clinical Efficacy Exploration of Temperature-Sensitive Embolic Agent TACE Combined with HAIC and Targeted/Immunotherapy for Unresectable Primary Liver Cancer JOURNAL OF HEPATOCELLULAR CARCINOMA | 2026 | 10.2147/jhc.s572484 | No eligible comparative arm: Small retrospective case series of TACE-HAIC plus targeted/immunotherapy without an eligible control group. |
| 6 | Wu C. et al. Correction: TACE-HAIC versus HAIC combined with TKIs and ICIs for hepatocellular carcinoma with a high tumor burden—a propensity-score matching comparative study (Frontiers in Immunology, (2025), 16, (1664756), 10.3389/fimmu.2025.1664756) Frontiers in Immunology | 2026 | 10.3389/fimmu.2025.1769802 | Ineligible publication type: Correction notice rather than a primary comparative study. |
| 7 | Liu et al. TACE-HAIC Plus Targeted Therapy and Immunotherapy for HCC with Tumor Thrombus and Arteriovenous Fistula JOURNAL OF HEPATOCELLULAR CARCINOMA | 2025 | 10.2147/jhc.s567414 | Ineligible comparator: TACE-HAIC plus targeted therapy/ICI was compared with TACE alone; the control lacked the prespecified systemic backbone. |
| 8 | Peng et al. Clinical efficacy of hepatic arterial infusion chemotherapy combined with transhepatic arterial embolization plus lenvatinib and tislelizumab or transarterial chemoembolization combined with lenvatinib plus tislelizumab in the treatment of advanced hepatocellular carcinoma FRONTIERS IN ONCOLOGY | 2025 | 10.3389/fonc.2025.1665348 | Ineligible intervention: Experimental locoregional regimen used HAIC plus transarterial embolization (TAE), not the prespecified TACE-HAIC intervention. |
| 9 | Yin L. et al. TACE-HAlC combined with donafenib and immune checkpoint inhibitors for BCLC stage C HCC patients (THEME study): A retrospective IPTW adjusted cohort study. Journal of Clinical Oncology | 2025 | 10.1200/jco.2025.43.16_suppl.4090 | Ineligible comparator: TACE-HAIC plus targeted therapy/ICI was compared with targeted-immunotherapy alone rather than a TACE- or HAIC-based control. |
| 10 | Komatsu et al. Utilizing the Conversion Concept Based on Disease Status Following Atezolizumab Plus Bevacizumab Treatment for Hepatocellular Carcinoma WORLD JOURNAL OF SURGERY | 2025 | 10.1002/wjs.70061 | Outcome/subgroup not separately extractable: Multiple post-systemic locoregional interventions were analyzed without a separately extractable prespecified TACE-HAIC versus TACE/HAIC comparison. |
| 11 | Yan H.-T. et al. MRI-Based Quantification of Intratumoral Heterogeneity Predicts Response in Hepatocellular Carcinoma Treated with Atezolizumab-Bevacizumab and TACE/HAIC Academic Radiology | 2025 | 10.1016/j.acra.2025.06.025 | No eligible comparative arm: Imaging/prediction study; no eligible comparator-specific treatment-effect analysis. |
| 12 | Hou et al. Efficacy of transarterial chemoembolization-hepatic arterial infusion chemotherapy combined with targeted therapy and immunotherapy in hepatocellular carcinoma with portal vein tumor thrombosis ONCOLOGY LETTERS | 2025 | 10.3892/ol.2025.15109 | Overlapping/superseded cohort: Patient cohort overlapped with the later Hou 2026 report from the same center/time window; the later report was retained to avoid double counting. |
| 13 | Deng W. et al. The safety and efficacy of tyrosine kinase inhibitors and programmed cell death protein- 1 inhibitors combined with HAIC/TACE in the treatment of recurrent unresectable hepatocellular carcinoma BMC Cancer | 2025 | 10.1186/s12885-025-14185-x | Ineligible intervention: Evaluated HAIC- or TACE-based targeted/PD-1 regimens; no TACE-HAIC intervention arm. |
| 14 | Wu et al. The Combination of D-TACE-HAIC, Lenvatinib, and PD-1 Inhibitors Shows Significant Clinical Efficacy in Patients with Unresectable Hepatocellular Carcinoma CANCER MANAGEMENT AND RESEARCH | 2025 | 10.2147/cmar.s481242 | Ineligible comparator: TACE-HAIC plus lenvatinib/PD-1 inhibitor was compared with TACE plus sorafenib, not the prespecified comparable targeted/ICI backbone. |
| 15 | Zhang L. et al. Efficacy and safety of transcatheter arterial chemoembolization followed by hepatic arterial infusion chemotherapy combined with TKI and PD-1 inhibitors as first-line treatment for advanced hepatocellular carcinoma Nan fang yi ke da xue xue bao = Journal of Southern Medical University | 2024 | 10.12122/j.issn.1673-4254.2024.09.24 | Ineligible comparator: Both groups received TACE-HAIC, but the comparator omitted PD-1/ICI therapy. |
| 16 | Chen et al. Clinical Effectiveness and Safety of Transarterial Chemoembolization: Hepatic Artery Infusion Chemotherapy Plus Tyrosine Kinase Inhibitors With or Without Programmed Cell Death Protein-1 Inhibitors for Unresectable Hepatocellular Carcinoma-A Retrospective Study ANNALS OF SURGICAL ONCOLOGY | 2024 | 10.1245/s10434-024-15933-2 | Ineligible comparator: Compared TACE-HAIC+TKI+PD-1 with TACE-HAIC+TKI; comparator lacked the prespecified ICI component. |
| 17 | Huang Z. et al. Atezolizumab and bevacizumab plus transarterial chemoembolization and hepatic arterial infusion chemotherapy for patients with high tumor burden unresectable hepatocellular carcinoma: A multi-center cohort study International Immunopharmacology | 2024 | 10.1016/j.intimp.2024.112711 | No eligible comparative arm: Single cohort receiving atezolizumab/bevacizumab plus TACE-HAIC; no eligible control arm. |
| 18 | Huang et al. PD-L1 inhibitor versus PD-1 inhibitor plus bevacizumab with transvascular intervention in unresectable hepatocellular carcinoma CLINICAL AND EXPERIMENTAL MEDICINE | 2024 | 10.1007/s10238-024-01415-y | Ineligible comparator: Both groups received TACE-HAIC; the study compared different systemic regimens rather than TACE-HAIC versus a single-locoregional-modality control. |
| 19 | Pang et al. Real-world efficacy and safety of TACE-HAIC combined with TKIs and PD-1 inhibitors in initially unresectable hepatocellular carcinoma INTERNATIONAL IMMUNOPHARMACOLOGY | 2024 | 10.1016/j.intimp.2024.112492 | No eligible comparative arm: Single-arm real-world study of TACE-HAIC plus TKIs and PD-1 inhibitors. |
| 20 | Zhao et al. Transarterial chemoembolization (TACE)-hepatic arterial infusion chemotherapy (HAIC) combined with PD-1 inhibitors plus lenvatinib as a preoperative conversion therapy for nonmetastatic advanced hepatocellular carcinoma: a single center experience TRANSLATIONAL CANCER RESEARCH | 2024 | 10.21037/tcr-24-93 | No eligible comparative arm: Single-center conversion-treatment experience without an eligible control arm. |
| 21 | Diao L. et al. Hepatic arterial infusion chemotherapy combined with lenvatinib and PD-1 inhibitors versus lenvatinib and PD-1 inhibitors for HCC refractory to TACE Journal of Gastroenterology and Hepatology (Australia) | 2024 | 10.1111/jgh.16463 | Ineligible intervention: Compared HAIC+lenvatinib+ICI with lenvatinib+ICI; no TACE-HAIC intervention arm. |
| 22 | Li et al. [Study on the comparison of postoperative liver injury caused by hepatic arterial perfusion chemotherapy combined with targeted immunotherapy with hepatic arterial chemoembolization combined with targeted immunotherapy for intermediate-and advanced-stage liver cancer]. Zhonghua gan zang bing za zhi = Zhonghua ganzangbing zazhi = Chinese journal of hepatology | 2023 | 10.3760/cma.j.cn501113-20230827-00070 | Ineligible intervention: Compared HAIC-based with TACE-based targeted-immunotherapy; no TACE-HAIC intervention arm. |
| 23 | Long et al. Comparable Clinical Outcomes Between Transarterial Chemoembolization or Hepatic Arterial Infusion Chemotherapy Combined with Tyrosine Kinase Inhibitors and PD-1 Inhibitors in Unresectable Hepatocellular Carcinoma JOURNAL OF HEPATOCELLULAR CARCINOMA | 2023 | 10.2147/jhc.s436211 | Ineligible intervention: Compared TACE-based with HAIC-based targeted/PD-1 therapy; no TACE-HAIC intervention arm. |
| 24 | Yu B. et al. Tyrosine Kinase Inhibitors Plus Anti-PD-1 Antibodies with Hepatic Arterial Infusion Chemotherapy or Transarterial Chemoembolization for Unresectable Hepatocellular Carcinoma Journal of Hepatocellular Carcinoma | 2023 | 10.2147/jhc.s431917 | Ineligible intervention: Compared HAIC-based with TACE-based targeted/PD-1 therapy; no TACE-HAIC intervention arm. |
| 25 | Yuan Y. et al. TACE-HAIC combined with targeted therapy and immunotherapy versus TACE alone for hepatocellular carcinoma with portal vein tumour thrombus: a propensity score matching study International Journal of Surgery | 2023 | 10.1097/js9.0000000000000256 | Ineligible comparator: TACE-HAIC plus targeted/ICI therapy was compared with TACE alone. |
| 26 | Hiraoka A. et al. Does first-line treatment have prognostic impact for unresectable HCC?—Atezolizumab plus bevacizumab versus lenvatinib Cancer Medicine | 2023 | 10.1002/cam4.4854 | Ineligible intervention: Systemic-treatment comparison without the prespecified TACE-HAIC intervention. |
| 27 | Gao et al. Supplementary Material for: Survival Benefit of Combined Systemic and Locoregional Therapy in Hepatocellular Carcinoma with Portal Vein Tumor Thrombus: A Propensity Score-Matched Analysis Figshare | 2026 | 10.6084/m9.figshare.32021223 | Ineligible publication type: Supplementary-material record corresponding to another report, not an independent primary study. |
| 28 | Yin L. et al. TACE-HAIC combined with donafenib and immunotherapy for hepatocellular carcinoma (HCC) with portal vein tumour thrombus (PVTT): A retrospective analysis Journal of Clinical Oncology | 2024 | Journal of Clinical Oncology | No eligible comparative arm: Single-arm retrospective TACE-HAIC plus donafenib/PD-1 study. |
| 29 | Huang P. et al. FOLFOX-HAIC/TACE combined with lenvatinib plus tislelizumab as a conversion therapy in initial unresectable hepatocellular carcinoma: A retrospective real-world study. Journal of Clinical Oncology | 2025 | 10.1200/jco.2025.43.16_suppl.e16201 | Ineligible publication type: Conference abstract only; no eligible full peer-reviewed report. |
| 30 | Li R. et al. Comparison of liver injury after transcatheter arterial chemoembolization and hepatic arterial infusion chemotherapy for intermediate and advanced hepatocellular carcinoma Annals of Oncology | 2023 | 10.1016/j.annonc.2023.10.781 | Ineligible intervention: Compared TACE-based with HAIC-based targeted/ICI therapy; no TACE-HAIC intervention arm. |
| 31 | Yang Y. et al. Analysis of the Safety and Effectiveness of Lenvatinib + TACE-HAIC + PD-1 Inhibitor for Intermediate and Advanced Hepatocellular Carcinoma Letters in Drug Design and Discovery | 2024 | 10.2174/1570180820666230601113529 | Ineligible comparator: TACE-HAIC+lenvatinib+PD-1 was compared with TACE+sorafenib, an ineligible systemic backbone. |
| 32 | Liu et al. The efficacy of TACE-HAIC combined with targeted and immunotherapy in hepatocellular carcinoma with portal vein tumor thrombosis Zenodo | 2025 | 10.5281/zenodo.14722470 | Ineligible publication type: Zenodo-only/non-peer-reviewed report without an eligible full journal publication. |
| 33 | Qiu J. et al. 1005P Atezolizumab plus bevacizumab combined with transarterial chemoembolization and hepatic arterial infusion chemotherapy for unresectable hepatocellular carcinoma with high tumor burden Annals of Oncology | 2023 | 10.1016/j.annonc.2023.09.2149 | Ineligible publication type: Conference abstract only (1005P). |
| 34 | Huang X.-Y. et al. Sequential hepatic artery infusion chemotherapy and transarterial chemoembolization combined with anti-vascular endothelial growth factor antibody/tyrosine kinase inhibitors and immune checkpoint inhibitors (quadruple therapy) as first-line therapy for advanced hepatocellular carcinoma: A single-center retrospective cohort study. Journal of Clinical Oncology | 2025 | 10.1200/jco.2025.43.16_suppl.e16180 | No eligible comparative arm: Single-cohort quadruple-therapy study without an eligible comparator. |
| 35 | Zang M. et al. COMPARISON OF LIVER FUNCTION INJURY IN INTERMEDIATE AND ADVANCED HEPATOCELLULAR CARCINOMA PATIENTS TREATED WITH HEPATIC ARTERIAL INFUSION CHEMOTHERAPY VERSUS TRANSCATHETER ARTERIAL CHEMOEMBOLIZATION COMBINED WITH IMMUNE CHECKPOINT INHIBITORS AND TYROSINE KINASE INHIBITORS Hepatology | 2024 | 10.1097/hep.0000000000001077 | Ineligible intervention: Compared HAIC-based with TACE-based targeted/ICI therapy; no TACE-HAIC intervention arm. |
| 36 | Xia et al. Efficacy and Safety Analysis of Local-Systemic Combined Therapy Versus Systemic Therapy Alone in TACE-Resistant Hepatocellular Carcinoma Patients HEPATITIS MONTHLY | 2026 | 10.5812/hepatmon-168214 | Outcome/subgroup not separately extractable: Local-systemic group pooled heterogeneous local modalities (modified TACE, HAIC, or IMRT); the prespecified TACE-HAIC comparison was not separately extractable. |
| 37 | Zeng Y. et al. Effectiveness and safety of camrelizumab plus rivoceranib combined with TACE with or without HAIC in first-line treatment of unresectable hepatocellular carcinoma Annals of Oncology | 2025 | 10.1016/j.annonc.2025.08.2112 | Overlapping/superseded cohort: Prospective 19-center cohort overlapped with another report/cohort already represented in the review; excluded to avoid double counting. |
| 38 | The Efficacy and Safety of Donafenib Plus PD-1/L1 Monoclonal Antibodies Plus Transarterial Chemoembolization (TACE) or Hepatic Arterial Infusion Chemotherapy (HAIC) as First-line Treatment for Unresectable Hepatocellular carcinomaA Multicenter, Retrospective Clinical Study clinicaltrials.gov | 2026 | clinicaltrials.gov | Ineligible publication type: Clinical-trial registry record without an eligible completed full report; planned comparison was TACE versus HAIC rather than TACE-HAIC. |
| 39 | Xue W. et al. Hepatic arterial infusion chemotherapy combined with transcatheter chemoembolization, camrelizumab, and apatinib for BCLC stage C hepatocellular carcinoma: A retrospective study ESMO Open | 2026 | 10.1016/j.esmoop.2026.107917 | No eligible comparative arm: Single-arm retrospective study of HAIC+TACE+camrelizumab+apatinib. |
| 40 | Yang X.S. et al. Hepatic arterial infusion chemotherapy combined with transcatheter chemoembolization, camrelizumab, and apatinib for hepatocellular carcinoma with portal vein tumor thrombosis: A retrospective study ESMO Open | 2026 | 10.1016/j.esmoop.2026.107915 | No eligible comparative arm: Single-arm retrospective study of HAIC+TACE+camrelizumab+apatinib. |
| 41 | Study of the Safety, Clinical Efficacy and Prognostic Factors of TACE and/or HAIC Combined With Molecular-targeted Therapy and Immunotherapy for Hepatocellular Carcinoma clinicaltrials.gov | 2026 | clinicaltrials.gov | Ineligible publication type: Clinical-trial registry record without completed peer-reviewed comparative results. |
| 42 | Hepatic Arterial Infusion Chemotherapy Plus Sintilimab and Bevacizumab Versus Transarterial Chemoembolization Plus Sintilimab and Bevacizumab for Intermediate-Stage Hepatocellular Carcinoma Beyond the Up-To-Seven Criteria：A Prospective, Randomized, Controlled Trial clinicaltrials.gov | 2026 | clinicaltrials.gov | Ineligible publication type: Clinical-trial registry/protocol record without completed peer-reviewed results. |
| 43 | MATCH-001: A Multicenter, Open-Label, Randomized Controlled Trial of Hepatic Arterial Infusion Chemotherapy Followed by Transarterial Chemoembolization Combined With Apatinib and Camrelizumab in Patients With Intermediate and Advanced Hepatocellular Carcinoma clinicaltrials.gov | 2026 | clinicaltrials.gov | Ineligible publication type: Clinical-trial registry/protocol record without completed peer-reviewed results. |
| 44 | Zhang Z. et al. HAIC-ICI-VEGF/TKIs versus TACE-ICI-VEGF/TKIs for Unresectable Hepatocellular Carcinoma with Tumors ≥10 cm: A Target Trial Emulation Study SSRN | 2026 | 10.2139/ssrn.6580846 | Ineligible publication type: Preprint/SSRN report; unpublished/non-peer-reviewed study under the final eligibility criteria. |
| 45 | Efficacy and Safety of Sequential FOLFOX-HAIC Followed by TACE Combined With Sintilimab Plus Bevacizumab as Neoadjuvant Therapy, Followed by Surgical Resection, Versus Direct Surgical Resection in Patients With Resectable Hepatocellular Carcinoma and High-Risk Recurrence Factors: A Single-Center, Open-Label, Two-Arm, Randomized Study clinicaltrials.gov | 2026 | clinicaltrials.gov | Ineligible population: Neoadjuvant study in resectable HCC with high-risk recurrence features, outside the prespecified unresectable population. |
| 46 | Toripalimab Plus Bevacizumab Combined Three Intra-arterial Therapies (Transarterial Chemoembolization, Hepatic Artery Infusion Chemotherapy, or Transarterial Chemoembolization Plus Hepatic Artery Infusion Chemotherapy) for Unresectable Hepatocellular Carcinoma: a Phase 2, Multicenter Randomised Non-comparative Trial clinicaltrials.gov | 2026 | clinicaltrials.gov | No eligible comparative arm: Randomized non-comparative phase 2/registry study; no eligible comparative treatment-effect arm. |
| 47 | TACE Versus HAIC, Combined With PD-1 Inhibitors and Lenvatinib for Unresectable Hepatocellular Carcinoma: a Multicenter, Prospective, Observational Cohort Study clinicaltrials.gov | 2026 | clinicaltrials.gov | Ineligible publication type: Clinical-trial registry record without completed peer-reviewed results; planned comparison did not include a TACE-HAIC intervention arm. |
| 48 | He B. et al. Systemic treatment patterns and survival outcomes for hepatocellular carcinoma - A multicenter real-world study Annals of Oncology | 2025 | 10.1016/j.annonc.2025.10.190 | Outcome/subgroup not separately extractable: Large real-world systemic-therapy cohort with heterogeneous transarterial treatments; the prespecified comparator-specific data were not separately extractable. |
| 49 | HAIC vs. TACE Combined With ICIs and TKIs in Intermediate-to-Advanced HCC: A Multicenter Study on Liver Injury and Survival clinicaltrials.gov | 2025 | clinicaltrials.gov | Ineligible publication type: Clinical-trial registry record without completed peer-reviewed results. |
| 50 | Zhang H. et al. Comparison of the efficacy and safety of HAIC combined with camrelizumab and apatinib versus TACE in the treatment of intermediate-advanced HCC Hepatology International | 2025 | 10.1007/s12072-025-10854-y | Ineligible intervention: Compared HAIC-based targeted/immunotherapy with a TACE-based regimen; no TACE-HAIC intervention arm. |
| 51 | Zhou Z. et al. Hepatic arterial infusion chemotherapy combined with donafenib and tislelizumab versus transcatheter chemoembolization alone for hepatocellular carcinoma: A propensity score matching study. Journal of Clinical Oncology | 2025 | 10.1200/jco.2025.43.16_suppl.4094 | Ineligible publication type: Conference abstract only; additionally, the comparator was TACE alone. |
| 52 | Lenvatinib, Sintilimab, and Drug-Eluting Beads Transarterial Chemoembolization With or Without Hepatic Arterial Infusion Chemotherapy for Hepatocellular Carcinoma >7 cm With Portal Vein Tumor Thrombus: A Multicenter, Randomized Controlled Trial clinicaltrials.gov | 2025 | clinicaltrials.gov | Ineligible publication type: Ongoing clinical-trial registry/protocol; no completed peer-reviewed results. |
| 53 | Diao L. et al. FOLFOX-HAIC combined with lenvatinib and immune checkpoint inhibitors for hepatocellular carcinoma after the occurrence of TACE refractoriness: analysis of efficacy and safety Journal of Interventional Radiology (China) | 2024 | 10.3969/j.issn.1008-794x.2024.06.005 | Ineligible intervention: HAIC+lenvatinib+ICI after TACE refractoriness; no TACE-HAIC intervention arm. |
| 54 | Chen L. et al. Hepatic arterial infusion chemotherapy versus transarterial chemoembolization combined with tyrosine kinase inhibitors and camrelizumab for unresectable hepatocellular carcinoma: a comparative study Journal of Interventional Radiology (China) | 2024 | 10.3969/j.issn.1008-794x.2024.05.015 | Ineligible intervention: Compared HAIC-based with TACE-based targeted/ICI therapy; no TACE-HAIC intervention arm. |
| 55 | Atezolizumab Plus Bevacizumab Versus Sintilimab Plus Bevacizumab With Transarterial Chemoembolization and Hepatic Arterial Infusion Chemotherapy in Unresectable Hepatocellular Carcinoma: A Multicenter Real-World Study clinicaltrials.gov | 2023 | clinicaltrials.gov | Ineligible publication type: Clinical-trial registry record; planned comparison focused on systemic regimens given with TACE-HAIC rather than an eligible locoregional comparator. |
| 56 | Perioperative Sintilimab Plus Bevacizumab Biosimilar and Transarterial Embolization, Hepatic Artery Infusion Chemotherapy (TACE-HAIC) for Hepatocellular Carcinoma Patients With Portal Vein Tumor Thrombus (PVTT): A Phase-2 Clinical Trial clinicaltrials.gov | 2023 | clinicaltrials.gov | Ineligible publication type: Phase 2 clinical-trial registry/protocol without completed peer-reviewed comparative results. |
| 57 | Yin L. et al. Chemoembolization plus hepatic arterial infusion chemotherapy combined with donafenib, anti-PD-1 antibody for unresectable hepatocellular carcinoma (uHCC): A retrospective analysis Journal of Clinical Oncology | 2023 | Journal of Clinical Oncology | No eligible comparative arm: Single-arm retrospective TACE-HAIC plus donafenib/PD-1 study. |
| 58 | HAIC Combined With Lenvatinib and Tislelizumab Versus D-TACE Combined With Lenvatinib and Tislelizumab in Advanced Unresectable Hepatocellular Carcinoma clinicaltrials.gov | 2022 | clinicaltrials.gov | Ineligible intervention: Compared HAIC+lenvatinib+tislelizumab with D-TACE+lenvatinib+tislelizumab; no TACE-HAIC intervention arm. |
| 59 | TACE-HAIC Combined With Targeted Therapy and Immunotherapy Versus TACE Alone for Hepatocellular Carcinoma With Portal Vein Tumor Thrombus: A Propensity Score Matching Study clinicaltrials.gov | 2022 | clinicaltrials.gov | Ineligible comparator: TACE-HAIC plus targeted/ICI therapy was compared with TACE alone. |
| 60 | TACE/HAIC Combined With Lenvatinib and Sintilimab in Neoadjuvant Therapy for Patients With Potentially High Recurrence Risk After Liver Cancer Resection: a Prospective, Randomized, Controlled Clinical Study clinicaltrials.gov | 2022 | clinicaltrials.gov | Ineligible population: Neoadjuvant trial in patients planned for liver resection/high recurrence risk, outside the prespecified unresectable population. |
| 61 | Liu C. et al. Efficacy analysis of comprehensive therapy of immune checkpoint inhibitors (ICIs) and tyrosine kinase inhibitors (TKIs) combined with HAIC or TACE in unresectable hepatocellular carcinoma (uHCC) Liver Cancer | 2022 | 10.1159/000528570 | Ineligible intervention: Evaluated HAIC- or TACE-based targeted/ICI regimens; no TACE-HAIC intervention arm. |
| 62 | Evaluation Effectiveness and Safety of (cTACE or DEB-TACE + FOLFOX Regimen HAIC) Combined With Camrelizumab and Apatinib Mesylas in the Treatment of Advanced Hepatocellular Carcinoma clinicaltrials.gov | 2020 | clinicaltrials.gov | No eligible comparative arm: Prospective exploratory single-arm registry study of TACE/DEB-TACE+HAIC plus camrelizumab/apatinib. |
| 63 | 匡嘉文 et al. TACE-HAIC联合仑伐替尼和PD-1抑制剂四联治疗Ⅲa期肝癌的疗效 中华肝脏外科手术学电子杂志 | 2025 | 中华肝脏外科手术学电子杂志 | Ineligible comparator: TACE-HAIC+lenvatinib+PD-1 was compared with TACE-HAIC+lenvatinib; comparator lacked an ICI. |
| 64 | 王远鹏 et al. TACE联合HAIC加靶免“四联”疗法对中晚期肝细胞癌的疗效分析 肝胆胰外科杂志 | 2025 | 肝胆胰外科杂志 | Ineligible comparator: Control regimen was internally inconsistent within the article (reported as TACE alone in one section and TACE+lenvatinib+sintilimab in another), so the comparator could not be reliably classified. |
| 65 | 程明亮 et al. 肝动脉灌注化疗联合信迪利单抗+贝伐珠单抗注射液对不可切除肝癌患者的临床疗效 肝脏 | 2024 | 肝脏 | Ineligible intervention: Compared HAIC-based with TACE-based sintilimab/bevacizumab therapy; no TACE-HAIC intervention arm. |
| 66 | 吕嘉晖 et al. TACE+HAIC联合卡瑞利珠单抗与甲磺酸阿帕替尼治疗不可切除肝细胞癌的前瞻性真实世界研究 腹部外科 | 2025 | 腹部外科 | Overlapping/superseded cohort: Patient population overlapped with another cohort already represented in the review; excluded to avoid double counting. |
| 67 | 邱林彬 et al. HAIC与TACE联合卡瑞利珠单抗及仑伐替尼治疗中晚期原发性肝癌患者的近期疗效比较 医学理论与实践 | 2025 | 医学理论与实践 | Ineligible intervention: Compared HAIC-based with TACE-based camrelizumab/lenvatinib therapy; no TACE-HAIC intervention arm. |
| 68 | 廖全辉 et al. 肝动脉化疗栓塞加肝动脉灌注化疗对中晚期肝癌的疗效观察 深圳中西医结合杂志 | 2023 | 深圳中西医结合杂志 | Ineligible population: Study population did not meet the prespecified population eligibility criteria, as confirmed during manual adjudication. |
| 69 | 陶杰 et al. 联合卡瑞利珠单抗和仑伐替尼的肝动脉灌注化疗与肝动脉化疗栓塞对不可切除肝细胞癌的疗效对比 重庆医学 | 2023 | 重庆医学 | Ineligible intervention: Compared HAIC-based with TACE-based camrelizumab/lenvatinib therapy; no TACE-HAIC intervention arm. |
| 70 | 刘演 et al. 基于TACE的多联治疗在中晚期肝细胞癌中疗效差异的回顾性研究 肝胆外科杂志 | 2023 | 肝胆外科杂志 | Outcome/subgroup not separately extractable: Multiple TACE-based combination levels were pooled; the small quadruple-therapy subgroup did not provide a separately extractable eligible comparator-specific effect. |
| 71 | 杨宇光 et al. 仑伐替尼、PD-1抑制剂联合肝动脉栓塞灌注化疗治疗不可切除肝癌疗效 中华肝脏外科手术学电子杂志 | 2023 | 中华肝脏外科手术学电子杂志 | Ineligible comparator: TACE-HAIC+lenvatinib+PD-1 was compared with sorafenib+TACE rather than the prespecified comparable targeted/ICI backbone. |
| 72 | 彭紫谦 et al. 肝癌肝动脉灌注化疗栓塞术联合靶向免疫治疗后乙肝病毒再激活的临床评价 中国医院药学杂志 | 2025 | 中国医院药学杂志 | Ineligible intervention: HBV-reactivation study of TACE plus targeted/immunotherapy without a TACE-HAIC intervention arm. |
| 73 | 钟桂红 et al. 经桡动脉TACE+HAIC联合免疫靶向治疗对术后肝癌并门静脉癌栓患者的影响 医学理论与实践 | 2024 | 医学理论与实践 | Ineligible comparator: Compared TACE-HAIC alone with TACE-HAIC plus targeted/immunotherapy; control lacked the prespecified systemic backbone. |
| 74 | 吴新桦 et al. TACE-HAIC联合靶免对比HAIC联合靶免在不可切除肝细胞癌转化治疗中的疗效 重庆医科大学 | 2024 | 10.27674/d.cnki.gcyku.2024.001463 | Ineligible publication type: Master’s/doctoral thesis; grey literature was not included in the final quantitative synthesis. |
| 75 | 肝动脉灌注化疗栓塞术联合肝细胞靶向免疫治疗原发性肝癌的临床疗效 | 2025 | 63 | Ineligible intervention: No prespecified TACE-HAIC intervention arm, as confirmed during manual adjudication. |
| 76 | 经导管肝动脉化疗栓塞术和肝动脉灌注化疗联合阿替利珠单抗和贝伐珠单抗治疗晚期不可切除肝癌的疗效分析 | 2026 | 10.3969/j.issn.1002-1671.2026.04.027 | No eligible comparative arm: Single-arm study, as confirmed during manual adjudication. |
| 77 | 匡天佐 et al. 经肝动脉化疗栓塞术-肝动脉灌注化疗术-靶向-免疫四联治疗在肝癌转化治疗中的应用研究 医学理论与实践 | 2025 | 医学理论与实践 | Ineligible comparator: TACE-HAIC+targeted/immune therapy was compared with targeted-immunotherapy alone rather than a TACE- or HAIC-based control. |
| 78 | 陈磊 et al. 肝动脉灌注化疗序贯肝动脉栓塞术联合靶向-免疫治疗中晚期肝癌临床疗效观察 山西医药杂志 | 2025 | 山西医药杂志 | Ineligible intervention: Used HAIC followed by transarterial embolization (TAE), not the prespecified TACE-HAIC intervention. |
| 79 | 严伟 et al. HAIC-FOLFOX与TACE分别联合靶向免疫药物在 治疗中晚期肝细胞癌中的疗效对比 当代介入医学电子杂志 | 2026 | 当代介入医学电子杂志 | Ineligible intervention: Compared HAIC-FOLFOX+targeted/immune therapy with TACE+targeted/immune therapy; no TACE-HAIC intervention arm. |
| 80 | 姚俊宇 et al. 三种不同介入方式联合靶向及免疫治疗初始不可切除肝细胞癌的疗效分析 广西医科大学 | 2025 | 10.27038/d.cnki.ggxyu.2025.000949 | Ineligible publication type: Master’s/doctoral thesis; grey literature was not included in the final quantitative synthesis. |
| 81 | 杜楠 et al. 肝癌TACE还是HAIC? 介入放射学杂志 | 2024 | 介入放射学杂志 | Ineligible publication type: Narrative review rather than an original comparative study. |
| 82 | 王爱华 et al. 肝动脉灌注化疗栓塞术联合肝细胞靶向免疫治疗对原发性巨块型肝癌的治疗效果 河北医药 | 2024 | 河北医药 | Ineligible intervention: Evaluated TACE plus targeted/immunotherapy versus TACE plus targeted therapy; no HAIC/TACE-HAIC intervention. |
| 83 | 和蓓露 et al. 乙型肝炎病毒相关肝细胞癌患者临床特征及生存分析(2018—2020年) 国际流行病学传染病学杂志 | 2024 | 国际流行病学传染病学杂志 | No eligible comparative arm: Prognostic cohort with heterogeneous treatments; no prespecified comparator-specific TACE-HAIC treatment comparison. |
| 84 | 李辉艳 et al. FOLFOX-HAIC联合靶免治疗中晚期肝癌的疗效观察 右江民族医学院 | 2023 | 10.27908/d.cnki.gymzy.2023.000039 | Ineligible publication type: Master’s/doctoral thesis; grey literature was not included in the final quantitative synthesis. |
| 85 | 温耀鸿 et al. 多纳非尼联合PD-1抑制剂及血管介入治疗不可切除肝细胞癌的疗效与安全性 南方医科大学 | 2023 | 10.27003/d.cnki.gojyu.2023.000848 | Ineligible publication type: Master’s/doctoral thesis; grey literature was not included in the final quantitative synthesis. |
| 86 | 陈旭颖 et al. HAIC改良方案联合仑伐替尼、卡瑞利珠单抗治疗BCLC C期原发性肝细胞癌的临床研究 三峡大学 | 2023 | 10.27270/d.cnki.gsxau.2023.000691 | Ineligible publication type: Master’s/doctoral thesis; study compared HAIC dosing schedules within TACE-HAIC-based therapy rather than the prespecified comparator. |
| 87 | 吴印涛 et al. 肝动脉载药微球栓塞灌注化疗(D-TACE-HAIC)联合仑伐替尼、PD-1抑制剂治疗合并门静脉癌栓的肝癌临床疗效观察 中文科技期刊数据库（全文版）医药卫生 | 2023 | 中文科技期刊数据库（全文版）医药卫生 | Ineligible comparator: D-TACE-HAIC+lenvatinib+PD-1 was compared with sorafenib rather than an eligible TACE- or HAIC-based targeted/ICI control. |

Supplementary Figure Legends

Supplementary Figure S1. Leave-one-out sensitivity analyses for the TACE-based comparison. (A) Overall survival (OS); (B) progression-free survival (PFS); (C) objective response rate (ORR); (D) disease control rate (DCR); (E) complete response (CR).

Supplementary Figure S2. Leave-one-out sensitivity analyses for the HAIC-based comparison. (A) Overall survival (OS); (B) progression-free survival (PFS); (C) objective response rate (ORR); (D) disease control rate (DCR); (E) complete response (CR).

Supplementary Figure S3. TACE-based sensitivity analyses after exclusion of Li 2025. (A) Overall survival (OS); (B) progression-free survival (PFS); (C) objective response rate (ORR); (D) disease control rate (DCR); (E) complete response (CR).

Supplementary Figure S4. HAIC-based survival sensitivity analyses after exclusion of studies judged at serious overall risk of bias by ROBINS-I. (A) Overall survival (OS); (B) progression-free survival (PFS).
